# Role of Human Mesenchymal Stem Cells and Derived Extracellular Vesicles in Reducing Sensory Neuron Hyperexcitability and Pain Behaviors in Murine Osteoarthritis

**DOI:** 10.1002/art.42353

**Published:** 2022-12-28

**Authors:** Minji Ai, William E. Hotham, Luke A. Pattison, Qingxi Ma, Frances M.D. Henson, Ewan St. John Smith

**Affiliations:** ^1^ Department of Veterinary Medicine University of Cambridge UK; ^2^ Department of Surgery and Department of Medicine University of Cambridge UK; ^3^ Department of Pharmacology University of Cambridge UK; ^4^ Department of Surgery University of Cambridge UK

## Abstract

**Objective:**

Mesenchymal stem/stromal cells (MSCs) and MSC‐derived extracellular vesicles (MSC‐EVs) have been reported to alleviate pain in patients with knee osteoarthritis (OA). We undertook this study to determine whether MSCs and/or MSC‐EVs reduce OA pain through influencing sensory neuron excitability in OA joints.

**Methods:**

We induced knee OA in adult male C57BL/6J mice through destabilization of the medial meniscus (DMM) surgery. Mice were sorted into 4 experimental groups with 9 mice per group as follows: unoperated sham, untreated DMM, DMM plus MSC treatment, and DMM plus MSC‐EV treatment. Treated mice received either MSCs at week 14 postsurgery or MSC‐EVs at weeks 12 and 14 postsurgery. Mouse behavior was evaluated by digging and rotarod tests and the Digital Ventilated Cage system. At week 16, mouse knee joints were harvested for histology, and dorsal root ganglion (DRG) neurons were isolated for electrophysiology. Furthermore, we induced hyperexcitability in DRG neurons in vitro using nerve growth factor (NGF) then treated these neurons with or without MSC‐EVs and evaluated neuron excitability.

**Results:**

MSC‐ and MSC‐EV–treated DMM‐operated mice did not display pain‐related behavior changes (in locomotion, digging, and sleep) that occurred in untreated DMM‐operated mice. The absence of pain‐related behaviors in MSC‐ and MSC‐EV–treated mice was not the result of reduced joint damage but rather a lack of knee‐innervating sensory neuron hyperexcitability that was observed in untreated DMM‐operated mice. Furthermore, we found that NGF‐induced sensory neuron hyperexcitability is prevented by MSC‐EV treatment (*P* < 0.05 versus untreated NGF‐sensitized neurons when comparing action potential threshold).

**Conclusion:**

MSCs and MSC‐EVs may reduce pain in OA by direct action on peripheral sensory neurons.

## INTRODUCTION

Osteoarthritis (OA) is a debilitating musculoskeletal disease affecting ~300 million people worldwide (1). Chronic pain is the primary OA symptom, and poorly managed OA pain can limit joint function (2), reduce quality of life (e.g., compromised sleep quality [3]), and lead to long‐term disability (4). Unfortunately, current pharmacologic treatments for OA pain (e.g., nonsteroidal antiinflammatory drugs and opioids) fail to provide sufficient pain relief and are often associated with unwanted side effects following chronic use (5). Therefore, managing OA pain remains challenging and requires the development of disease‐specific analgesics to address this unmet clinical need.

Peripheral nociceptive input is a major contributor to OA pain as demonstrated by reduced pain in OA patients following intraarticular injections of the local anesthetic lidocaine (6) and loss of peripheral nociceptive input following total knee replacement, although pain persists in some patients (7). Moreover, in rodents, specific inhibition of nociceptor activity with the quaternary anesthetic QX‐314 ameliorated early OA pain (8), and we have previously shown that inflammatory joint pain drives changes in behavior that can be reversed through chemogenetic inhibition of knee‐innervating sensory neurons in mice (9). Furthermore, in the monoiodoacetate model of OA in rats, extracellular electrophysiologic recordings of knee‐innervating neurons show that they become sensitized early after disease onset (from day 3) and that this is maintained, whereas bone‐innervating afferents only become sensitized later in the disease course (day 28) (10).

Studies have identified several key molecules that are thought to be involved in OA pain development and can be developed as disease‐specific pain targets. For example, nerve growth factor (NGF) was identified as a target for potential pain treatment for OA because its expression was elevated in a murine OA model (11), and treatment with soluble NGF receptor tropomyosin‐related kinase receptor A (TrkA) (11), treatment with an anti‐NGF antibody (12), and inhibition of the TrkA receptor (13) can all effectively suppress pain‐like behavior in rodent OA models. Moreover, a number of anti‐NGF antibodies have demonstrated clinical efficacy in managing OA pain in patients, but the risk of causing rapidly progressive OA has thus far prevented their clinical application (14).

In search of disease‐modifying anti‐OA drugs, mesenchymal stem/stromal cell (MSC) therapy has emerged as a promising treatment, with clinical trials demonstrating pain relief and improved joint function in OA patients (15). MSCs exert a strong immunomodulatory effect in OA‐affected joints through the paracrine mechanisms, which lead to analgesic and anticatabolic effects in OA‐affected joints (16). However, a further possibility exists that they may directly alter nociceptive input, which could contribute to the pain relief experienced by those with OA. However, the direct link between MSCs and nociception in OA remains unexplored; that is, do MSCs affect neuron excitability?

Despite promising outcomes, the clinical use of MSCs faces safety concerns, such as potential tumorigenicity (17). Therefore, extracellular vesicles secreted by MSCs (MSC‐EVs) have been proposed as an alternative to MSCs for treating OA. Indeed, increasing evidence has attributed the therapeutic effects of MSCs to their paracrine secretion, especially EVs (18, 19, 20). EVs are small, membrane‐bound vesicles (30–2,000 nm in diameter) that are secreted into the extracellular space by cells, including MSCs (21). Within EVs, there is a rich profile of biomolecules, including proteins, lipids, and nucleic acids, which have strong immunomodulating and chondroprotective properties (22). Preclinical studies show that MSC‐EVs derived from various sources (e.g., adipose tissue, bone marrow, and umbilical cord MSCs) exert a similar therapeutic effect on their source cells in different OA models, such as inhibiting joint inflammation and promoting cartilage repair (23). However, the analgesic effects of MSC‐EVs in OA remain unknown.

In this study, we aimed to determine to what extent either MSCs or MSC‐EVs provide analgesia by studying their impact on nociception in OA‐affected joints. We hypothesized that MSCs and MSC‐EVs would improve OA pain via direct modulation of sensory neurons innervating the joint.

## MATERIALS AND METHODS

### Animals

**Figure 1:**
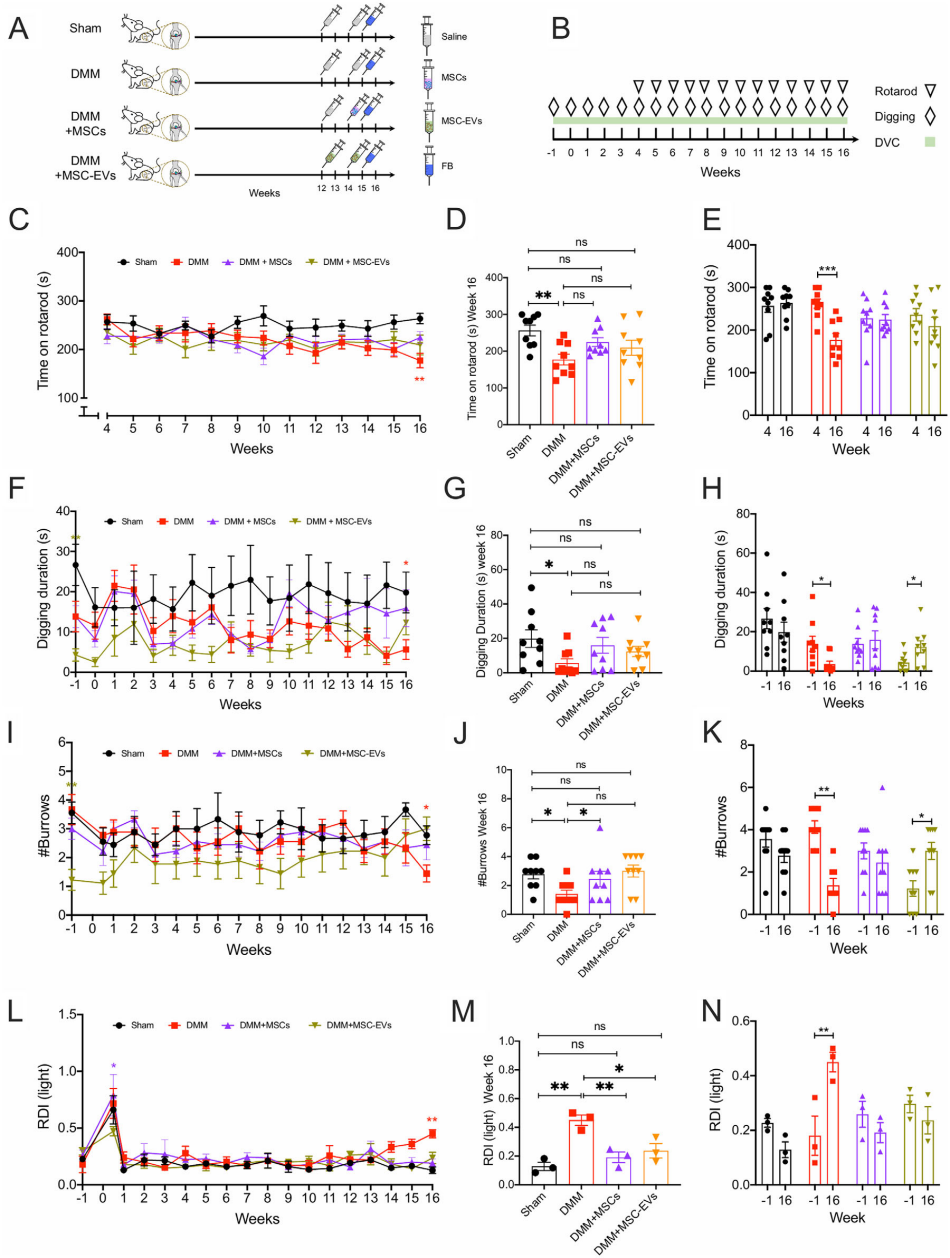
**A**, Mice (n = 36) were assigned 1:1:1:1 to sham, destabilization of medial meniscus (DMM) surgery without treatment, DMM with mesenchymal stem/stromal cell (MSC) treatment, or DMM with MSC‐derived extracellular vesicle (MSC‐EV) treatment. Mice in all groups were injected with fast blue (FB) at week 15 postsurgery, and mouse knee joints were harvested at week 16. **B**, Timeline of behavior tests and time mice spent in Digital Ventilated Cages (DVCs). **C–E**, Time series comparison of mouse groups from week 4 to week 16 (**C**), between‐group comparison at week 16 (**D**), and within‐group comparison between week 4 and week 16 (**E**) of time mice spent on rotarod. **F–N**, Time series comparison of mouse groups from presurgery to week 16 (**F**, **I**, **L**), between‐group comparison at week 16 (**G**, **J**, **M**), and within‐group comparison between presurgery and week 16 (**H**, **K**, **N**) of time mice spent digging (**F–H**), number of burrows mice dug (**I–K**), and regularity disruption index (RDI) values during lights‐on periods (**L–N**). Symbols represent individual mice. Dots or bars with whiskers show the mean ± SEM. * = *P* < 0.05; ** = *P* < 0.01; *** = *P* < 0.001 by two‐way analysis of variance (ANOVA) followed by Tukey's post hoc test for time series comparisons, one‐way ANOVA followed by Tukey's post hoc test for between‐group comparisons, or two‐way ANOVA followed by Holm‐Sidak multiple comparisons test for within‐group comparisons. ns = no significant difference. Color figure can be viewed in the online issue, which is available at http://onlinelibrary.wiley.com/doi/10.1002/art.42353/abstract.

All animal experiments were regulated under the Animals (Scientific Procedures) Act 1986, Amendment Regulations 2012, following ethical review by the University of Cambridge Animal Welfare and Ethical Review Body. A total of 36 male C57BL/6J mice ages 10–12 weeks (Charles River) were used for in vivo study. Mice were assigned into 1 of 4 experimental groups of 9 mice each as follows: 1) sham, 2) destabilization of the medial meniscus (DMM), 3) DMM plus MSC treatment, or 4) DMM plus MSC‐EV treatment (Figure 1A). All mice were housed in groups of 3 in separate Digital Ventilated Cages (DVCs) (cage model GM500; Tecniplast) with standard water and food supply during the experiment period.

### DMM surgery

DMM surgery was performed as previously described (24), and a detailed description of the surgery is provided in the Supplementary Methods available on the *Arthritis & Rheumatology* website at https://onlinelibrary.wiley.com/doi/10.1002/art.42353. Mice were allowed to recover in a 37°C chamber (20% oxygen) (Tecniplast) with welfare checks every 15 minutes for 1 hour until fully alert with no sign of lameness before being returned to their home cages. No inclusion/exclusion criteria were set, and no experimental units/data points were excluded from analysis.

### Knee injections

MSCs (2 × 10^4^ in 6 μl; Lonza) were injected into the stifle joint of DMM‐operated mice at 14 weeks postsurgery. MSC‐EVs (6 μl) derived from 2 × 10^4^ MSCs were injected into the stifle joint of DMM‐operated mice at 12 weeks and 14 weeks (see Supplementary Methods and Supplementary Figure 1, available at https://onlinelibrary.wiley.com/doi/10.1002/art.42353, for MSC culture, MSC‐EV harvest, and characterization). As a control, 6 μl of 0.9% saline was injected into the stifle joint of mice in the untreated DMM and sham groups at 12 and 14 weeks. To label knee‐innervating neurons, 1.5 μl of retrograde tracer fast blue (2% weight/volume in 0.9% saline; Polysciences) was injected into the operated stifle joints 7 days prior to euthanization. All injections were made under anesthesia.

### DVC system

DVCs were installed on a standard DVC rack (Tecniplast) with external electronic sensors and 12 uniformly distributed, contactless electrodes underneath each cage. Animal locomotion activity (referred to as activity in this article) was monitored by capacitance changes in the electrodes caused by animal movement and computed as previously described (25). Weekly regularity disruption index (RDI) during the lights‐on period was computed to capture irregular animal activity patterns as previously described (26). Data were processed and computed on the DVC analytics platform (Tecniplast).

### Rotarod

Mouse locomotion and coordination were carried out weekly using a rotarod apparatus (model 47600; Ugo Basile) starting 4 weeks after surgery (Figure 1B) (27). Mice were placed on the rotarod at a constant speed of 4 revolutions per minute for 1 minute before entering an accelerating testing mode (4–40 rpm in 5 minutes). Total time spent on the rotarod and the speed at the time the mice fell from the rotarod or after 2 passive rotations were recorded. The same protocol was used to train mice 1 day before the first test.

### Digging

The digging test was carried out weekly as previously described (28); a detailed description is provided in the Supplementary Methods, https://onlinelibrary.wiley.com/doi/10.1002/art.42353. Digging duration (time mice spent displacing bedding material using paws) and the number of dig sites (burrows) produced during the testing period were analyzed by 3 experimenters who were blinded with regard to the conditions in a random order (36% of videos scored by 2 experimenters, R^2^ correlation between scores = 0.95).

### Dorsal root ganglion (DRG) neuron isolation and culture

Lumbar DRGs (L2–L5) were collected postmortem, and DRG neurons were isolated and cultured as previously described (9); a detailed description of the isolation method is provided in the Supplementary Methods, https://onlinelibrary.wiley.com/doi/10.1002/art.42353.

### In vitro coculture of DRG neurons and MSC‐EVs

Lumbar DRG (L2–L5) neurons from nonoperated mice (n = 4) were isolated and cultured as above, or with addition of mouse NGFβ (100 ng/ml) (29). After 24 hours, medium was replaced without NGFβ, with 100 ng/ml NGFβ, or with NGF plus MSC‐EV (10^6^/ml). Neurons were then cultured for another 16 to 24 hours before electrophysiology recordings.

### Electrophysiology

Whole‐cell, patch‐clamp electrophysiology was performed on fast blue–labeled DRG neurons isolated from the 4 mouse groups and DRG neurons in the in vitro coculture groups. A detailed description of electrophysiology is given in the Supplementary Methods, https://onlinelibrary.wiley.com/doi/10.1002/art.42353.

### Histology

Safranin O–fast green histologic staining was performed on operated knee joints from each mouse group. A detailed description of tissue processing and staining is presented in the Supplementary Methods, https://onlinelibrary.wiley.com/doi/10.1002/art.42353. Images from at least 3 sections of each mouse joint and joints from 7–9 mice in each mouse group were included for analysis. Images obtained were scored by 2 experimenters who were blinded to the conditions using the Osteoarthritis Research Society International (OARSI) scoring system (30).

### Statistical analysis

All data are presented as the mean ± SEM. Two‐way analysis of variance (ANOVA) with Tukey's post hoc test was used for 4‐group comparisons across time series. Two‐way ANOVA with Holm‐Sidak multiple comparisons test was used for within‐ and between‐group comparisons. One‐way ANOVA with Tukey's post hoc test was used for comparisons across multiple groups. Detailed statistical tests are described in individual figure legends. Statistical analysis and graph generation were performed using GraphPad Prism software version 8.0.

## RESULTS

### Prevention of pain‐related behavior changes in DMM‐operated mice treated with MSCs and MSC‐EVs

We observed that untreated DMM‐operated mice spent significantly less time on the rotarod than unoperated mice in the sham group at week 16 (mean ± SEM 177.1 ± 14.77 seconds in DMM group versus 256.4 ± 14.6 seconds in sham group; *P* = 0.0008 by one‐way ANOVA followed by Tukey's post hoc test) (Figures 1C and D). In contrast, time spent on the rotarod by DMM‐operated mice treated with MSCs or MSC‐EVs was not significantly different compared to mice in the sham group at week 16 (mean ± SEM 224.8 ± 11.88 seconds in DMM plus MSC group, *P* = 0.06 versus sham; 219.4 ± 20.57 seconds in DMM plus MSC‐EV group, *P* = 0.09 versus sham) (Figures 1C and D). Additionally, compared to untreated DMM‐operated mice, DMM‐operated mice treated with MSCs (*P* = 0.13 versus untreated DMM) and DMM‐operated mice treated with MSC‐EVs (*P* = 0.7 versus untreated DMM) did not spend a significantly longer time on the rotarod at 16 weeks (Figure 1D). Within groups, untreated DMM‐operated mice spent significantly less time on the rotarod at week 16 than at week 4 (mean ± SEM 262.2 ± 10.89 seconds at week 4, *P* = 0.0005 by two‐way ANOVA followed by Holm‐Sidak multiple comparisons test) (Figure 1E). However, there was no within‐group difference in the sham group (mean ± SEM 263.7 ± 10.73 seconds at week 4, *P* = 0.99) or the treated DMM groups (227.7 ± 15.91 seconds at week 4 in MSC group, *P* = 0.99; 236 ± 14.58 seconds at week 4 in MSC‐EV group, *P* = 0.54) (Figure 1E).

We reported previously that mice with joint pain spend less time digging burrows than healthy mice, and thus the digging behavior of mice can be considered an ethologically relevant pain assay (28). In line with the rotarod test, we observed that untreated DMM‐operated mice, but not MSC‐ and MSC‐EV–treated DMM‐operated mice, spent significantly less time digging than mice in the sham group at week 16 (mean ± SEM digging time 19.79 ± 5.07 seconds in sham group; 5.59 ± 2.45 seconds in untreated DMM group, *P* = 0.03 versus sham by one‐way ANOVA followed by Tukey's post hoc test; 15.87 ± 4.59 seconds in DMM plus MSC group, *P* = 0.89 versus sham; 12.32 ± 3.03 seconds in DMM plus MSC‐EV group, *P* = 0.46 versus sham) (Figures 1F and G). Consistent with digging time observations, untreated DMM‐operated mice, but not MSC‐ and MSC‐EV–treated DMM‐operated mice, dug significantly fewer burrows than mice in the sham group at week 16 (mean ± SEM 2.77 ± 0.32 burrows in sham group; 1.4 ± 0.26 burrows in untreated DMM group, *P* = 0.02 versus sham by one‐way ANOVA followed by Tukey's post hoc test; 2.44 ± 0.53 burrows in DMM plus MSC group, *P* = 0.9 versus sham; 3 ± 0.40 burrows in DMM plus MSC‐EV group, *P* = 0.95 versus sham) (Figures 1I and J). Compared to untreated DMM‐operated mice, no significant improvement in digging duration was seen in DMM‐operated mice treated with MSCs (*P* = 0.27 versus untreated DMM) or MSC‐EVs (*P* = 0.62 versus untreated DMM) (Figure 1G). MSC‐treated DMM‐operated mice dug significantly more burrows than untreated DMM‐operated mice (*P* = 0.028), but there was no significant difference between MSC‐treated and MSC‐EV–treated DMM‐operated mice (*P* = 0.75) (Figure 1J).

However, an innate difference in digging behavior was observed among the 4 mouse groups. Prior to surgery, mice in the DMM plus MSC‐EV group exhibited a significantly lower digging duration (mean ± SEM digging time 4.25 ± 1.51 seconds in DMM plus MSC‐EV group versus 26.66 ± 5.16 seconds in sham group; *P* = 0.005 by one‐way ANOVA followed by Tukey's post hoc test) (Figure 1H) and dug fewer burrows than mice in the sham group (mean ± SEM 1.37 ± 0.37 burrows in DMM plus MSC‐EV group versus 3.55 ± 0.37 burrows in sham group; *P* = 0.03) (Figure 1K). Within groups, untreated DMM‐operated mice exhibited reduced digging duration at 16 weeks compared to presurgery (presurgery mean ± SEM digging time 13.84 ± 3.8 seconds, *P* = 0.05 by two‐way ANOVA followed by Holm‐Sidak multiple comparisons test) (Figure 1H) and dug fewer burrows (presurgery mean ± SEM 4.11 ± 0.78 burrows, *P* = 0.003) (Figure 1K), whereas mice in the sham group and MSC‐treated DMM‐operated mice had a similar digging duration at week 16 compared to presurgery (presurgery mean ± SEM digging time 26.66 ± 5.16 seconds in sham group, *P* = 0.58; 13.82 ± 2.7 seconds in DMM plus MSC group, *P* = 0.99) (Figure 1H) and dug a similar number of burrows compared to presurgery (presurgery mean ± SEM 3.55 ± 0.37 burrows in sham group, *P* = 0.39; 2.55 ± 0.47 burrows in DMM plus MSC group, *P* = 0.69) (Figure 1K). At 16 weeks, MSC‐EV treated DMM‐operated mice had an increase in digging duration (mean ± SEM digging time 12.32 ± 3 seconds at week 16, *P* = 0.04 compared to presurgery) (Figure 1H) and number of burrows (mean ± SEM 3.0 ± 0.40 burrows at week 16, *P* = 0.03 compared to presurgery) (Figure 1K).

Unlike the rotarod and digging tests, which can only be conducted at set times and involve experimenter intervention, the DVC system continuously monitors activity. As expected, mice exhibited a high level of activity during the lights‐off period compared to the lights‐on period (Supplementary Figure 2A, available at https://onlinelibrary.wiley.com/doi/10.1002/art.42353). However, bouts of increased irregular activity were seen in untreated DMM‐operated mice during the lights‐on period (i.e., sleep/rest period) in the last week of housing (Supplementary Figure 2B), suggesting a possible irregularity in the rest pattern of untreated DMM‐operated mice caused by pain, similar to the OA‐induced impact on sleep observed in humans (3). This pattern of irregular activity was computed as the RDI, a digital biomarker measuring such irregularity (26).

Increased RDI during the lights‐on period has been previously reported in mice with amyotrophic lateral sclerosis during the early symptomatic period (26). We found that untreated DMM‐operated mice developed a significantly higher lights‐on RDI than mice in the sham group at week 16 (mean ± SEM RDI 0.45 ± 0.036 in untreated DMM group versus 0.12 ± 0.028 in sham group; *P* = 0.006 by one‐way ANOVA followed by Tukey's post hoc test) (Figures 1L and M), suggesting a more perturbed rest pattern during the lights‐on period in untreated DMM‐operated mice. Such an increase in lights‐on RDI was not observed in MSC‐ or MSC‐EV–treated DMM‐operated mice at week 16 (mean ± SEM RDI 0.19 ± 0.036 in DMM plus MSC group, *P* = 0.48 versus sham; 0.23 ± 0.049 in DMM plus MSC‐EV group, *P* = 0.29 versus sham) (Figures 1L and M). Additionally, compared to untreated DMM‐operated mice, DMM‐operated mice treated with MSCs (*P* = 0.006 versus untreated DMM) and MSC‐EVs (*P* = 0.01 versus untreated DMM) had significantly lower lights‐on RDI values at week 16, but these RDI values were not significantly different compared to the sham group (Figure 1M). Similarly, within groups, the lights‐on RDI of untreated DMM‐operated mice at week 16 was also significantly higher than their presurgery level (presurgery mean ± SEM RDI 0.18 ± 0.07, *P* = 0.007 by two‐way ANOVA followed by Holm‐Sidak multiple comparisons test) (Figure 1N). This increase in RDI was not seen in treated DMM‐operated mice (presurgery mean ± SEM RDI 0.25 ± 0.04 in DMM plus MSC group, *P* = 0.32; 0.29 ± 0.03 in DMM plus MSC‐EV group, *P* = 0.36) (Figure 1N).

Together, these results suggest that MSC‐ and MSC‐EV–treated DMM‐operated mice did not develop the pain‐related behavior changes seen in untreated DMM‐operated mice.

### No improvement in joint damage in DMM‐operated mice treated with MSCs and MSC‐EVs

**Figure 2:**
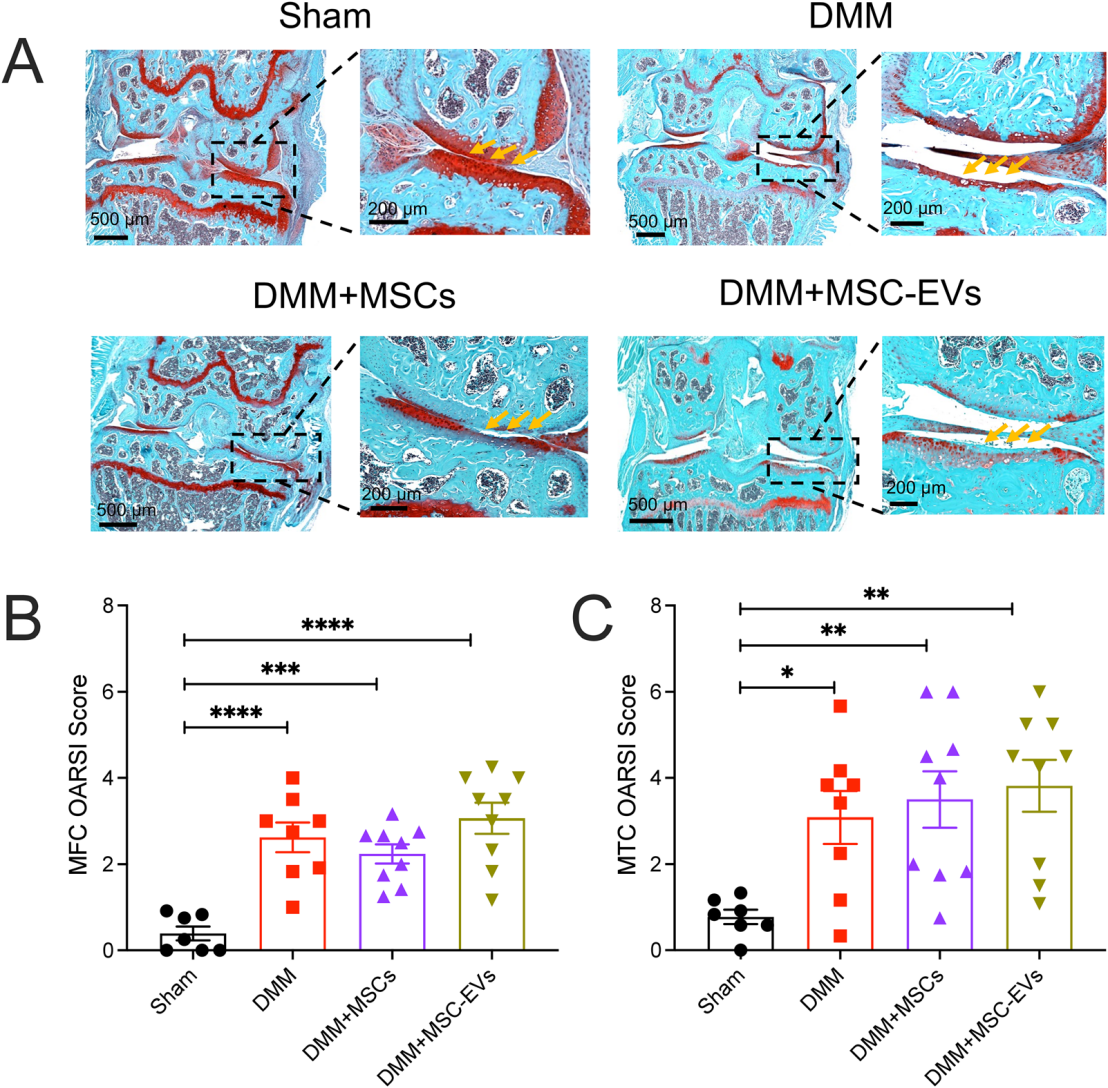
Histologic changes in DMM‐operated knee joints at 16 weeks after surgery in mice left untreated or treated with MSCs or MSC‐EVs compared to untreated, unoperated mice in the sham group. Knee joints were assessed for cartilage damage by staining with Safranin O–fast green (**A**) and Osteoarthritis Research Society International (OARSI) scores of the medial femoral condyle (MFC) (**B**) and medial tibial condyle (MTC) (**C**). **Yellow arrows** indicate intact cartilage in the sham group, whereas in DMM‐operated groups, cartilage loss is evident as reduced red staining. In **B** and **C**, symbols represent individual mice; bars with whiskers show the mean ± SEM. * = *P* < 0.05; ** = *P* < 0.01; *** = *P* < 0.001; **** = *P* < 0.0001 versus sham by one‐way ANOVA followed by Tukey's post hoc test. See Figure 1 for other definitions.

MSCs and MSC‐EVs are known to promote cartilage repair in OA joints and have been used as regenerative treatments for OA (19). Therefore, we next examined whether the lack of pain‐related behaviors in MSC‐ and MSC‐EV–treated DMM‐operated mice resulted from improved joint damage. We performed Safranin O–fast green staining on operated mouse knee joints to evaluate the cartilage damage in different groups and observed that mice from all 3 DMM‐operated groups presented with severe joint cartilage damage compared to unoperated mice in the sham group (Figure 2A).

Using the OARSI histologic grading system, we found that knee joints from all DMM‐operated mice had significantly higher scores than those from mice in the sham group on both the medial femoral condyle (mean ± SEM OARSI 0.39 ± 0.16 in sham group; 2.62 ± 0.34 in untreated DMM group, *P* < 0.0001 versus sham by one‐way ANOVA followed by Tukey's post hoc test; 2.24 ± 0.22 in DMM plus MSC group, *P* < 0.0001 versus sham; 3.06 ± 0.35 in DMM plus MSC‐EV group, *P* < 0.0001 versus sham) (Figure 2B) and the medial tibial condyle (0.77 ± 0.16 in sham group; 3.08 ± 0.61 in untreated DMM group, *P* = 0.004 versus sham; 3.5 ± 0.65 in DMM plus MSC group, *P* = 0.003 versus sham; 3.81 ± 0.6 in DMM plus MSC‐EV group, *P* = 0.0007 versus sham) (Figure 2C). Based on this analysis, our data suggest that injected MSCs and MSC‐EVs do not affect gross joint damage, and thus the observed change in pain‐related behaviors might result from an effect of MSCs and MSC‐EVs on sensory neurons innervating the knee joint.

### Normalization of knee‐innervating neuron hyperexcitability in DMM‐operated mice treated with MSCs and MSC‐EVs

**Figure 3:**
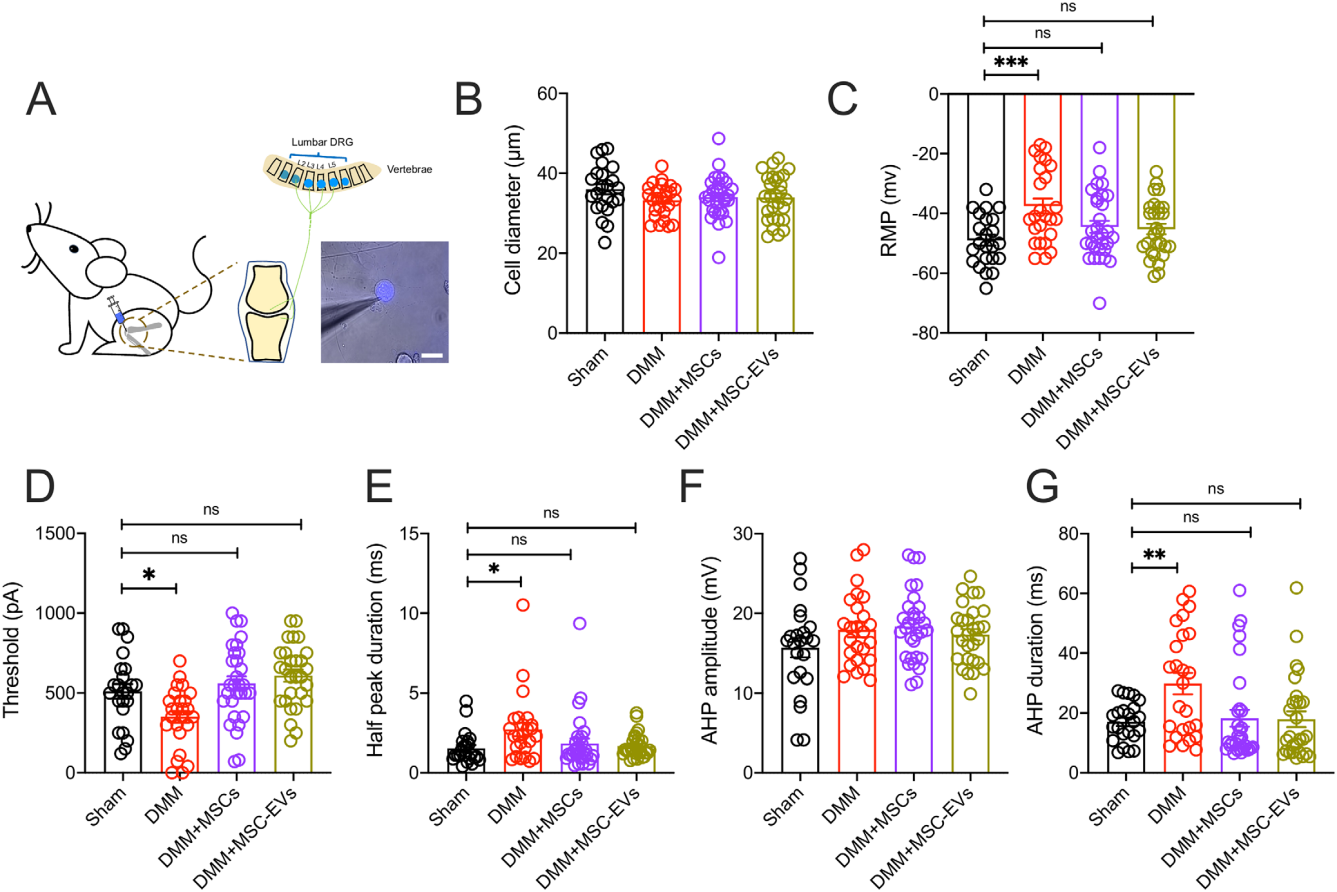
Characteristics of knee‐innervating dorsal root ganglion (DRG) neurons assessed in mice from each group using retrograde fast blue labeling of knee joints (**A**; bar = 50 μm), with electrophysiology studies comparing diameter (**B**), resting membrane potential (RMP) (**C**), threshold of electrical stimulus required for action potential (AP) firing (**D**), half peak duration (**E**), afterhyperpolarization (AHP) amplitude (**F**), and AHP duration (**G**). Symbols represent individual DRG neurons; bars with whiskers show the mean ± SEM. * = *P* < 0.05; ** = *P* < 0.01; *** = *P* < 0.001 versus sham by one‐way ANOVA followed by Tukey's post hoc test. ns = no significant difference (see Figure 1 for other definitions). Color figure can be viewed in the online issue, which is available at http://onlinelibrary.wiley.com/doi/10.1002/art.42353/abstract.

We have previously shown that knee‐innervating DRG sensory neuron excitability increases during acute joint inflammation and that inhibiting function of these neurons normalizes pain‐related behaviors (9, 28). Therefore, we injected fast blue into the operated mouse knee joints to label knee‐innervating neurons (Figure 3A). Cell bodies of these labeled neurons were then harvested after mice were killed 16 weeks postsurgery and identified by excitation with a 350 nm light source (Figure 3A). The excitability of neurons with similar diameters across groups was recorded by electrophysiology (Figure 3B). Table 1 presents the action potential (AP) properties of fast blue–labeled DRG neurons isolated from mice in the 4 mouse groups.

**Table 1:** Action potential properties of fast blue–labeled DRG neurons isolated from mice in each group in in vivo studies*

	Sham	Untreated DMM	DMM plus MSC	DMM plus MSC‐EV
	(n = 23)	(n = 25)	(n = 30)	(n = 28)
Diameter, μm	36.01 ± 2.17	33.2 ± 0.79	34.03 ± 1.00	33.91 ± 1.08
Resting membrane potential, mV	–48.96 ± 1.78	–37.52 ± 2.49†	–44.50 ± 2.03‡	–45.25 ± 1.78‡
Threshold, pA	509.60 ± 45.93	350.8 ± 37.52§	560 ± 43.56¶	607.5 ± 37.79¶
Half peak duration, msec	1.53 ± 0.20	2.73 ± 0.41#	1.83 ± 0.32	1.68 ± 0.14‡
Afterhyperpolarization duration, msec	17.07 ± 1.38	29.84 ± 3.54§	18.27 ± 2.82‡	17.93 ± 2.64**
Afterhyperpolarization amplitude, mV	15.69 ± 1.22	17.92 ± 0.90	18.34 ± 0.80	17.32 ± 0.70

* Values are the mean ± SEM. Each group included dorsal root ganglion (DRG) neurons from the knees of 6 mice. MSC = mesenchymal stem/stromal cell; MSC‐EV = MSC‐derived extracellular vesicle.

† *P* < 0.001 versus neurons from sham group by one‐way analysis of variance (ANOVA) followed by Tukey's post hoc test.

‡ *P* < 0.05 versus neurons from untreated destabilization of the medial meniscus (DMM) group by one‐way ANOVA followed by Tukey's post hoc test.

§ *P* < 0.01 versus sham by one‐way ANOVA followed by Tukey's post hoc test.

¶ *P* < 0.001 versus untreated DMM by one‐way ANOVA followed by Tukey's post hoc test.

# *P* < 0.05 versus sham by one‐way ANOVA followed by Tukey's post hoc test.

** *P* < 0.01 versus untreated DMM by one‐way ANOVA followed by Tukey's post hoc test.

We found that fast blue–positive neurons in untreated DMM‐operated mice had a significantly more depolarized resting membrane potential (RMP) than those from mice in the sham group (mean ± SEM RMP –37.52 ± 2.49 mV in untreated DMM group versus –48.96 ± 1.78 mV in sham group; *P* = 0.0009 by one‐way ANOVA followed by Tukey's post hoc test) (Figure 3C) and exhibited a significantly lower AP firing threshold (mean ± SEM 350.8 ± 37.52 pA in untreated DMM group versus 509.6 ± 45.93 pA in sham group; *P* = 0.03) (Figure 3D). No fast blue–labeled neurons displayed spontaneous firing in the sham group, whereas 9% of these in the untreated DMM group did. Additionally, fast blue–positive neurons from untreated DMM‐operated mice also had a longer half peak duration (HPD) than those from mice in the sham group (mean ± SEM HPD 2.72 ± 0.41 msec in untreated DMM group versus 1.53 ± 0.2 msec in sham group; *P* = 0.019) (Figure 3E) and a longer afterhyperpolarization (AHP) duration (mean ± SEM AHP 29.84 ± 3.54 msec in untreated DMM group versus 17.07 ± 1.38 msec in sham group; *P* = 0.006) (Figure 3G). These results suggested that DMM surgery induces knee‐innervating neuron hyperexcitability that likely underpins the changes in pain‐related behaviors observed.

When measuring the properties of fast blue–labeled neurons isolated from MSC‐ and MSC‐EV–treated DMM‐operated mice compared to those isolated from mice in the sham group, we observed no significant difference in RMP (mean ± SEM RMP –44.5 ± 2.03 mV in DMM plus MSC group, *P* = 0.29 versus sham; –45.25 ± 1.77 mV in DMM plus MSC‐EV group, *P* = 0.44 versus sham) (Figure 3B), or AP threshold (mean ± SEM 560 ± 43.53 pA in DMM plus MSC group, *P* = 0.71 versus sham; 607.5 ± 37.79 pA in DMM plus MSC‐EV group, *P* = 0.24 versus sham) (Figure 3C). Spontaneous firing was observed in 3% of fast blue–labeled neurons from MSC‐treated mice and 0% of those from MSC‐EV–treated mice; there was no significant difference in the proportion of spontaneously firing fast blue–labeled neurons across the 4 groups (*P* = 0.22 by Fisher's exact test). Moreover, the longer HPD and AHP durations seen in fast blue–labeled neurons isolated from untreated DMM‐operated mice were absent in neurons isolated from MSC‐ and MSC‐EV–treated mice (Figures 3E and G).

Because observed AP changes might result from changes in voltage‐gated ion channel function, we then analyzed the properties of macroscopic voltage‐gated inward and outward currents (Supplementary Figure 3, https://onlinelibrary.wiley.com/doi/10.1002/art.42353). Among knee neurons from mice in the untreated DMM and sham groups, little difference in normalized peak inward current (mean ± SEM 1.05 ± 0.12 in untreated DMM group versus 1 ± 0.08 in sham group; *P* = 0.7 by unpaired *t*‐test) (Supplementary Figure 3B) and outward current (mean ± SEM 1 ± 0.1 in untreated DMM group versus 1 ± 0.13 in sham group; *P* = 0.99 by unpaired *t*‐test) (Supplementary Figure 3D) was observed, and this was not investigated any further. Overall, these results suggest that knee‐innervating neurons are hyperexcitable in untreated DMM‐operated mice but not in MSC‐ and MSC‐EV–treated DMM‐operated mice.

### Normalization of NGF‐induced DRG neuron hyperexcitability by MSC‐EVs in vitro

**Figure 4:**
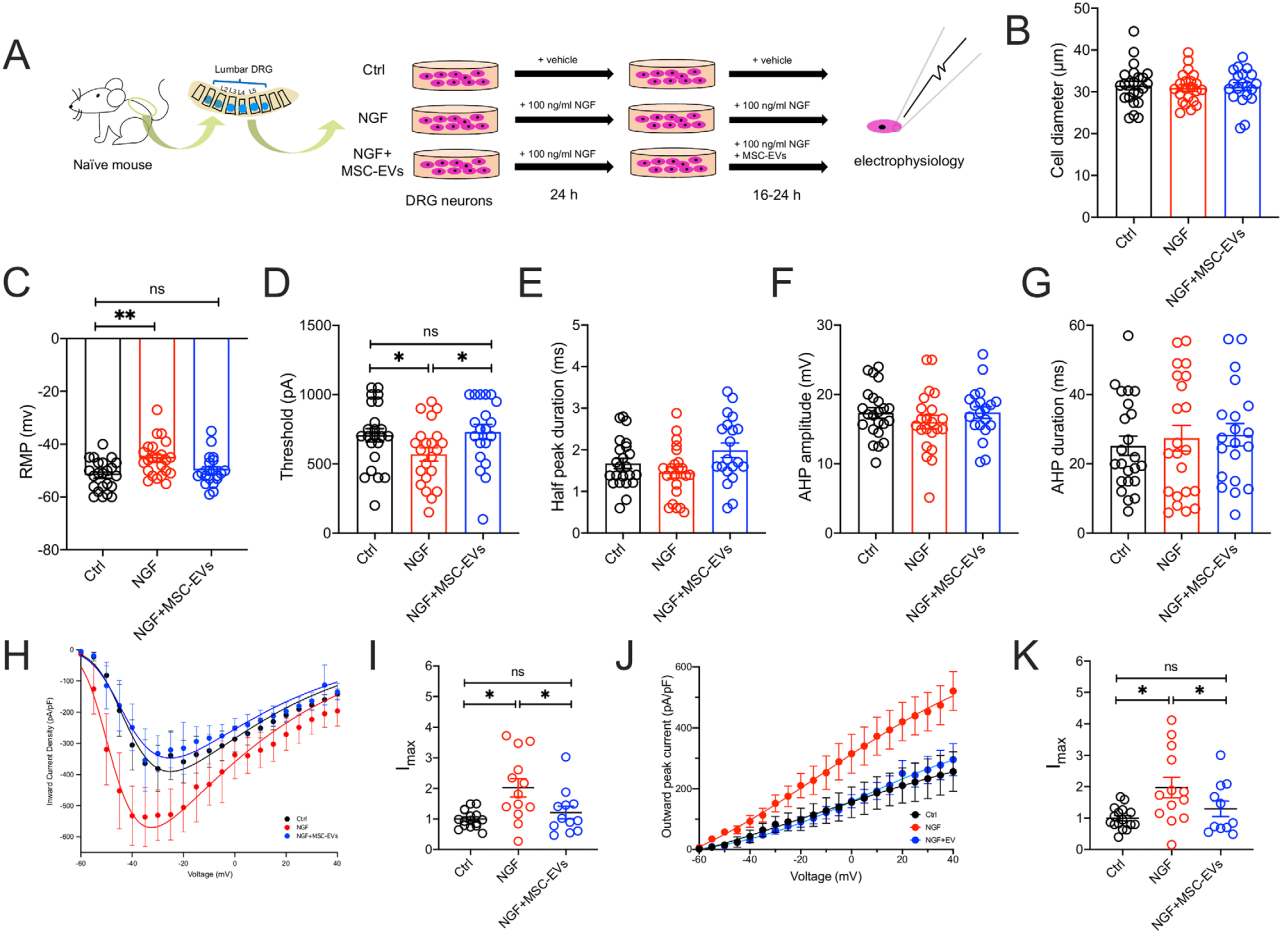
In vitro studies of DRG neurons performed in cultures with medium alone (control [Ctrl]), 100 ng/ml nerve growth factor β (NGFβ), or NGFβ plus MSC‐EVs (106/ml) for 16–24 hours (h) (**A)**, followed by electrophysiology studies comparing diameter (**B**), RMP (**C**), threshold of electrical stimulus required for AP firing (**D**), half peak duration (**E**), AHP amplitude (**F**), and AHP duration (**G**), as well as peak voltage‐gated inward (**H**) and outward (**J**) current density of DRG neurons normalized to cell capacitance in different conditions, and peak voltage‐gated inward (**I**) and outward (**K**) current normalized to maximum current density (I_max_) of control neurons. Symbols represent individual DRG neuron samples. Bars or horizontal lines with whiskers show the mean ± SEM. * = *P* < 0.05 and ** = *P* < 0.01 by one‐way ANOVA followed by Tukey's post hoc test. MSC‐EV = mesenchymal stem/stromal cell–derived extracellular vesicle (see Figure 3 for other definitions). Color figure can be viewed in the online issue, which is available at http://onlinelibrary.wiley.com/doi/10.1002/art.42353/abstract.

Based on the result that both MSC‐ and MSC‐EV–treated DMM‐operated mice did not develop pain‐related behaviors or knee‐innervating neuron hyperexcitability, we hypothesized that the MSC secretome, including MSC‐EVs, acts directly upon sensory neurons to normalize their hyperexcitability and in turn reduces pain. Based on this hypothesis, incubation of DRG sensory neurons with MSC‐EVs in vitro should be sufficient to normalize neuronal hyperexcitability. To test our hypothesis, we took advantage of the fact that NGF is involved in OA pain development in the DMM OA model (31), is present at enhanced levels at 16 weeks when pain‐related behaviors are observed (11), and directly induces DRG neuron hyperexcitability in vitro (32). Our initial experiments showed that coculturing DRG neurons with MSCs or conditioned medium containing MSC‐EVs had no significant impact on DRG neuron excitability (Supplementary Figure 4, https://onlinelibrary.wiley.com/doi/10.1002/art.42353). We therefore established 3 experimental groups as follows: 1) a control group with DRG neurons maintained in normal culture medium, 2) an NGF group with DRG neurons maintained in culture medium with addition of NGF, and 3) an NGF plus MSC‐EVs group in which DRG neurons were maintained in NGF‐added culture medium with addition of MSC‐EVs after 24 hours (Figure 4A). Table 2 presents the AP properties of the DRG neurons in the vitro groups.

**Table 2:** Action potential properties of mouse DRG neurons isolated from mice in each group in in vitro studies*

	Control	NGF	NGF plus MSC‐EV
	(n = 23)	(n = 23)	(n = 20)
Diameter, μm	31.51 ± 1.01	30.93 ± 0.78	31.24 ± 0.95
Resting membrane potential, mV	–51.78 ± 1.19	–45.48 ± 1.40†	–49.9 ± 1.30‡
Threshold, pA	706.5 ± 48.22	568.2 ± 47.39§	730 ± 54.34‡
Half peak duration, msec	1.67 ± 0.12	1.45 ± 0.13	1.98 ± 0.17‡
Afterhyperpolarization duration, msec	25.18 ± 2.77	17.39 ± 3.7	28.38 ± 3.25
Afterhyperpolarization amplitude, mV	17.33 ± 0.76	16.02 ± 0.96	17.39 ± 0.84

* Values are the mean ± SEM. Each group included seeded dorsal root ganglion (DRG) neurons isolated from 4 mice. MSC‐EV = mesenchymal stem/stromal cell–derived extracellular vesicle.

† *P* < 0.01 versus control neurons by one‐way analysis of variance (ANOVA) followed by Tukey's post hoc test.

‡ *P* < 0.05 versus nerve growth factor (NGF)–treated neurons by one‐way ANOVA followed by Tukey's post hoc test.

§ *P* < 0.05 versus control neurons by one‐way ANOVA followed by Tukey's post hoc test.

As expected, NGF‐treated DRG neurons had a significantly lower RMP than the control neurons (mean ± SEM RMP –45.48 ± 1.4 mV in NGF group versus –51.78 ± 1.19 mV in control group; *P* = 0.002 by one‐way ANOVA followed by Tukey's post hoc test) (Figure 4C) and exhibited a significantly lower AP threshold (mean ± SEM 568.2 ± 47.39 pA in NGF group versus 706.5 ± 48.22 pA in control group; *P* = 0.04) (Figure 4D). However, the RMP of DRG neurons in the NGF plus MSC‐EVs group was not significantly different from that of DRG neurons in the control group (mean ± SEM RMP –49.9 ± 1.3 mV in NGF plus MSC‐EV group, *P* = 0.059 versus control), and neither was the AP threshold (mean ± SEM 730 ± 54.34 pA in NGF plus MSC‐EV group, *P* = 0.94 versus control) (Figures 4C and D). Unlike what was observed in knee‐innervating DRG neurons isolated from DMM‐operated mice (Figures 3E and G), no significant change was seen in HPD or AHP duration in NGF‐treated DRG neurons; however, similar to results from analysis of knee‐innervating DRG neurons isolated from DMM‐operated mice, no difference was observed in the AHP amplitude (Figures 4E–G).

We again investigated whether the change in AP threshold might correlate with any change in the properties of voltage‐gated ion channel currents. We observed that DRG neurons in the NGF group, but not those in NGF plus MSC‐EV group, exhibited a significantly larger voltage‐gated inward current than control DRG neurons (mean ± SEM normalized peak inward current 1 ± 0.07 in control group; 2.01 ± 0.3 in NGF group, *P* = 0.003 versus control; 1.59 ± 0.27 in NGF plus MSC‐EV group, *P* = 0.74 versus control) (Figures 4H and I). In addition, voltage‐gated outward current amplitude was also larger in NGF‐treated neurons compared to control DRG neurons, but not significantly reversed in neurons from the NGF plus MSC‐EV group (mean ± SEM normalized peak outward current 1.01 ± 0.08 in control group; 1.81 ± 0.32 in NGF group, *P* = 0.03 versus control; 1.31 ± 0.25 in NGF plus MSC‐EV group, *P* = 0.6 versus control) (Figures 4J and K). Collectively, these results indicate that MSC‐EVs can normalize NGF‐induced DRG neuron excitability in vitro, but further work is required to establish the molecular mechanisms involved.

## DISCUSSION

Numerous preclinical (18, 19) and clinical studies (15) have demonstrated the potential use of MSCs and MSC‐EVs in treating OA, but the mechanism through which any pain‐relieving effects manifest has rarely been examined. Here, we found that hyperexcitability of knee‐innervating neurons in DMM‐operated mice was concomitant with observed behavior changes, and that intraarticular injection of either MSCs or MSC‐EVs reduced observed behavior changes and normalized knee‐innervating neuron hyperexcitability. These results suggest that primary afferent hyperexcitability is causal in DMM‐induced OA pain, which supports results of prior studies in rodents and humans showing the importance of primary afferent input in OA pain (33). This is the first study to directly measure the excitability of such afferents in the DMM mouse model.

In this study, we observed improved pain‐related behavior independent of any regenerative change in mouse OA knee joints following treatment with MSCs or MSC‐EVs. We used 3 methods to monitor mouse behavior: rotarod, digging assay, and activity monitoring. In the rotarod test, we observed a locomotion deficit when comparing untreated DMM‐operated mice to unoperated mice in the sham group at 16 weeks postsurgery and when comparing untreated DMM‐operated mice at week 16 postsurgery to themselves at week 4 postsurgery, consistent with previous reports (34, 35). However, such a deficit was not observed in MSC‐ or MSC‐EV–treated DMM‐operated mice. In the digging assay, reduced digging activity was seen in untreated DMM‐operated mice at week 16 but not in treated DMM‐operated mice or mice in the sham group.

Undeniably, innate mouse activity differences exist between different mouse groups. Mice in the DMM plus MSC‐EV group had lower digging activity than mice in other groups before surgery, but at week 16, the same group had similar digging activity to mice in the DMM plus MSC and sham groups, and higher digging activity than their presurgery level, which suggest that the observed difference in digging activity presurgery appears to have been compensated by repeated digging measurements over the 16‐week experimental period. While significant improvement in rotarod and behavior tests was not unanimously present in MSC‐ or MSC‐EV–treated DMM groups, the lack of difference between treated DMM groups and the sham group is sufficient to suggest behavioral improvement of treated DMM‐operated mice.

With activity monitoring, we discovered for the first time that OA mice display enhanced levels of irregular activity during resting periods as disease progresses (i.e., an increased RDI), similar to sleep disturbances seen in symptomatic OA patients, among whom 50–80% report reduced sleep quality, which is positively correlated with pain (3, 36). Such levels of irregular activity were not seen in treated DMM‐operated mice or mice in the sham group at week 16 or in any mice presurgery. Furthermore, MSC‐ and MSC‐EV–treated DMM‐operated mice showed reduced irregularity in their activity (lower RDI) than untreated DMM‐operated mice. These results indicate that both MSCs and MSC‐EVs normalize resting activity patterns in OA mice. Collectively, these data suggest that irregular behavior changes shown in the DMM‐operated mice were alleviated when the DMM‐operated mice were treated with either MSCs or MSC‐EVs (Figure 1). Moreover, the behavior normalization observed appears to be independent of gross improvement in joint histology (Figure 2), although we cannot rule out changes in synovitis or fibrosis, which were not analyzed in this study.

Sensory neuron sensitization is known to underlie the pain‐related behavior changes that occur in rat OA models (37), and sensory neuron hyperexcitability is also common to mouse and sheep models of joint pain (9, 28, 38). Thus, we performed electrophysiologic characterization of retrograde‐labeled, knee‐innervating neurons and observed depolarization of the RMP and lowering of the AP threshold in knee‐innervating neurons isolated from untreated DMM‐operated mice compared to those isolated from mice in the sham group, effects that were not observed in neurons isolated from DMM‐operated mice treated with MSCs or MSC‐EVs (Figure 3). This suggests that normalization of peripheral input may play a role in normalizing behavior. Despite this interesting observation, we acknowledge that normalization of peripheral sensory neuron excitability is unlikely to fully explain the observed behavior changes as both peripheral and central sensitization components contribute to OA pain; for example, sensitization of spinal nociceptive reflexes has been observed in a rat OA model (39). Whether the improved pain‐related behavior reported in this study is the result of changes to both peripheral and spinal nociceptive neuron activity remains unclear.

The normalization of peripheral sensory neuron excitability following MSC or MSC‐EV injection observed in this study might result from a direct action on sensory neurons and/or reduced nociceptive input/sensitization through modulation of surrounding cellular activity (e.g., reduced release of proinflammatory mediators by synoviocytes) (40). To address these potential mechanisms, we set up an in vitro model to test if MSC‐EVs directly alter sensory neuron activity. We induced hypersensitivity in naive mouse DRG neurons by incubating with NGF in vitro, which has a major role in OA pain (11) and induces DRG neuron hypersensitivity (41). As expected, NGF‐treated DRG neurons had a depolarized RMP and a lower AP threshold (Figures 4B and C), which coincubation with MSC‐EVs prevented. These results provide initial evidence that MSC‐EVs may normalize nociception in the OA joint through direct action on joint sensory neurons, but does not rule out an accompanying indirect effect.

However, a limitation of this study is that the in vitro analysis of the NGF‐treated DRG neurons did not fully replicate the changes observed in knee‐innervating neurons isolated from untreated DMM‐operated mice. The longer HPD and longer AHP duration seen in knee‐innervating neurons isolated from untreated DMM‐operated mice were not observed in NGF‐treated DRG neurons (Figures 4D and F), and knee‐innervating neurons from untreated DMM‐operated mice did not exhibit the larger voltage‐gated inward currents observed in NGF‐treated DRG neurons. Consequently, how MSC‐EVs modulate neuronal function may differ in vitro versus in vivo; nonetheless, data presented here establish models by which the modulatory mechanisms can be further investigated. It would also be of interest to investigate the effects of MSC‐EVs on the sensitizing effects of other mediators associated with OA pain, such as CCL2 (42, 43).

Overall, our study investigated changes in pain‐related behaviors and knee‐innervating sensory neuron function induced in a mouse DMM OA model and how these are altered by the administration of MSCs or MSC‐EVs. In doing so, we discovered that MSC‐EVs normalize sensory neuron hyperexcitability both in vivo and in vitro. These results open the possibility of using MSC‐EVs for chronic pain management. Future studies will focus on identifying molecular mechanisms involved in the analgesic effects observed.

## AUTHOR CONTRIBUTIONS

All the authors were involved in drafting the article or revising it critically for important intellectual content, and all authors approved the final version to be published. Dr. Smith had full access to all the data in the study and takes responsibility for the integrity of the data and the accuracy of the data analysis.

### Study conception and design

Ai, Pattison, Henson, Smith.

### Acquisition of the data

Ai, Hotham.

### Analysis and interpretation of the data

Ai, Pattison, Ma, Henson, Smith.

## Supporting information


Disclosure Form

**Appendix S1:** Supplementary Information

## References

[1] Safiri S, Kolahi A, Smith E , et al. Global, regional and national burden of osteoarthritis 1990–2017: a systematic analysis of the Global Burden of Disease Study 2017. Ann Rheum Dis 2020;79:819–28. 10.1136/annrheumdis-2019-216515 32398285

[2] McDonough CM , Jette AM . The contribution of osteoarthritis to functional limitations and disability [review]. Clin Geriatr Med 2010;26:387–99. 20699161 10.1016/j.cger.2010.04.001 PMC3529154

[3] Martinez R , Reddy N , Mulligan EP , et al. Sleep quality and nocturnal pain in patients with hip osteoarthritis. Medicine (Baltimore) 2019;98:e17464. 31593103 10.1097/MD.0000000000017464 PMC6799734

[4] Neogi T. The epidemiology and impact of pain in osteoarthritis. Osteoarthr Cartil 2013;21:1145–53. 10.1016/j.joca.2013.03.018 PMC3753584 23973124

[5] Moore N , Pollack C , Butkerait P . Adverse drug reactions and drug‐drug interactions with over‐the‐counter NSAIDs [review]. Ther Clin Risk Manag 2015;11:1061–75. 26203254 10.2147/TCRM.S79135 PMC4508078

[6] Eker HE , Cok OY , Aribogan A , et al. The efficacy of intra‐articular lidocaine administration in chronic knee pain due to osteoarthritis: a randomized, double‐blind, controlled study. Anaesth Crit Care Pain Med 2017;36:109–14. 27485803 10.1016/j.accpm.2016.05.003

[7] Beswick AD , Wylde V , Gooberman‐Hill R , et al. What proportion of patients report long‐term pain after total hip or knee replacement for osteoarthritis? A systematic review of prospective studies in unselected patients. BMJ Open 2012;2:e000435. 10.1136/bmjopen-2011-000435 PMC3289991 22357571

[8] Haywood AR , Hathway GJ , Chapman V . Differential contributions of peripheral and central mechanisms to pain in a rodent model of osteoarthritis. Sci Rep 2018;8:1–12. 29740093 10.1038/s41598-018-25581-8 PMC5940779

[9] Chakrabarti S , Pattison LA , Doleschall B , et al. Intraarticular adeno‐associated virus serotype AAV‐PHP.S–mediated chemogenetic targeting of knee‐innervating dorsal root ganglion neurons alleviates inflammatory pain in mice. Arthritis Rheumatol 2020;72:1749–58. 32418284 10.1002/art.41314

[10] Morgan M , Thai J , Nazemian V , et al. Changes to the activity and sensitivity of nerves innervating subchondral bone contribute to pain in late‐stage osteoarthritis. Pain 2022;163:390–402. 34108432 10.1097/j.pain.0000000000002355 PMC8756348

[11] McNamee KE , Burleigh A , Gompels LL , et al. Treatment of murine osteoarthritis with TrkAd5 reveals a pivotal role for nerve growth factor in non‐inflammatory joint pain. Pain 2010;149:386–92. 20350782 10.1016/j.pain.2010.03.002

[12] LaBranche TP , Bendele AM , Omura BC , et al. Nerve growth factor inhibition with tanezumab influences weight‐bearing and subsequent cartilage damage in the rat medial meniscal tear model. Ann Rheum Dis 2017;76:295–302. 27381034 10.1136/annrheumdis-2015-208913 PMC5264211

[13] Nwosu LN , Mapp PI , Chapman V , et al. Blocking the tropomyosin receptor kinase A (TrkA) receptor inhibits pain behaviour in two rat models of osteoarthritis. Ann Rheum Dis 2016;75:1246–54. 26286016 10.1136/annrheumdis-2014-207203 PMC4893148

[14] Vincent TL . Peripheral pain mechanisms in osteoarthritis [review]. Pain 2020;161:S138–46. 33090747 10.1097/j.pain.0000000000001923 PMC7434216

[15] Qu H , Sun S . Efficacy of mesenchymal stromal cells for the treatment of knee osteoarthritis: a meta‐analysis of randomized controlled trials. J Orthop Surg Res 2021;16:11. 33407686 10.1186/s13018-020-02128-0 PMC7789676

[16] Weiss ARR , Dahlke MH . Immunomodulation by mesenchymal stem cells (MSCs): mechanisms of action of living, apoptotic, and dead MSCs [review]. Front Immunol 2019;10:1191. 31214172 10.3389/fimmu.2019.01191 PMC6557979

[17] Barkholt L , Flory E , Jekerle V , et al. Risk of tumorigenicity in mesenchymal stromal cell–based therapies—bridging scientific observations and regulatory viewpoints. Cytotherapy 2013;15:753–9. 23602595 10.1016/j.jcyt.2013.03.005

[18] Cosenza S , Ruiz M , Toupet K , et al. Mesenchymal stem cells derived exosomes and microparticles protect cartilage and bone from degradation in osteoarthritis. Sci Rep 2017;7:16214. 29176667 10.1038/s41598-017-15376-8 PMC5701135

[19] Woo CH , Kim HK , Jung GY , et al. Small extracellular vesicles from human adipose‐derived stem cells attenuate cartilage degeneration. J Extracell Vesicles 2020;9:1735249. 32284824 10.1080/20013078.2020.1735249 PMC7144299

[20] Zhao X , Zhao Y , Sun X , et al. Immunomodulation of MSCs and MSC‐derived extracellular vesicles in osteoarthritis [review]. Front Bioeng Biotechnol 2020;8:575057. 33251195 10.3389/fbioe.2020.575057 PMC7673418

[21] EL Andaloussi S , Mäger I , Breakefield XO , et al. Extracellular vesicles: biology and emerging therapeutic opportunities [review]. Nat Rev Drug Discov 2013;12:347–57. 23584393 10.1038/nrd3978

[22] Dabrowska S , Andrzejewska A , Janowski M , et al. Immunomodulatory and regenerative effects of mesenchymal stem cells and extracellular vesicles: therapeutic outlook for inflammatory and degenerative diseases [review]. Front Immunol 2021;11:591065. 33613514 10.3389/fimmu.2020.591065 PMC7893976

[23] Kim KH , Jo JH , Cho HJ , et al. Therapeutic potential of stem cell‐derived extracellular vesicles in osteoarthritis: preclinical study findings [review]. Lab Anim Res 2020;36:10. 32322556 10.1186/s42826-020-00043-3 PMC7160998

[24] Glasson SS , Blanchet TJ , Morris EA . The surgical destabilization of the medial meniscus (DMM) model of osteoarthritis in the 129/SvEv mouse. Osteoarthritis Cartilage 2007;15:1061–9. 17470400 10.1016/j.joca.2007.03.006

[25] Pernold K , Iannello F , Low BE , et al. Towards large scale automated cage monitoring ‐ diurnal rhythm and impact of interventions on in‐cage activity of C57BL/6J mice recorded 24/7 with a non‐disrupting capacitive‐based technique. PLoS One 2019;14:e0211063. 30716111 10.1371/journal.pone.0211063 PMC6361443

[26] Golini E , Rigamonti M , Iannello F , et al. A non‐invasive digital biomarker for the detection of rest disturbances in the SOD1G93A mouse model of ALS. Front Neurosci 2020;14:896. 32982678 10.3389/fnins.2020.00896 PMC7490341

[27] Shiotsuki H , Yoshimi K , Shimo Y , et al. A rotarod test for evaluation of motor skill learning. J Neurosci Methods 2010;189:180–5. 20359499 10.1016/j.jneumeth.2010.03.026

[28] Chakrabarti S , Pattison LA , Singhal K , et al. Acute inflammation sensitizes knee‐innervating sensory neurons and decreases mouse digging behavior in a TRPV1‐dependent manner. Neuropharmacology 2018;143:49–62. 30240782 10.1016/j.neuropharm.2018.09.014 PMC6277850

[29] Lawrence GW , Zurawski TH , Dolly JO . Ca^2+^ signalling induced by NGF identifies a subset of capsaicin‐excitable neurons displaying enhanced chemo‐nociception in dorsal root ganglion explants from adult pirt‐GCaMP3 mouse. Int J Mol Sci 2021;22:2589. 33806699 10.3390/ijms22052589 PMC7961361

[30] Glasson SS , Chambers MG , Van Den Berg WB , et al. The OARSI histopathology initiative ‐ recommendations for histological assessments of osteoarthritis in the mouse. Osteoarthritis Cartilage 2010;18:S17–23. 10.1016/j.joca.2010.05.025 20864019

[31] Driscoll C , Chanalaris A , Knights C , et al. Nociceptive sensitizers are regulated in damaged joint tissues, including articular cartilage, when osteoarthritic mice display pain behavior. Arthritis Rheumatol 2016;68:857–67. 26605536 10.1002/art.39523 PMC4979655

[32] Zhang YH , Kays J , Hodgdon KE , et al. Nerve growth factor enhances the excitability of rat sensory neurons through activation of the atypical protein kinase C isoform, PKMζ. J Neurophysiol 2012;107:315–35. 21975456 10.1152/jn.00030.2011 PMC3349696

[33] Syx D , Tran PB , Miller RE , et al. Peripheral mechanisms contributing to osteoarthritis pain [review]. Curr Rheumatol Rep 2018;20:9. 29480410 10.1007/s11926-018-0716-6 PMC6599517

[34] Kojima S , Watanabe M , Asada K . Locomotor activity and histological changes observed in a mouse model of knee osteoarthritis. J Phys Ther Sci 2020;32:370–4. 32581428 10.1589/jpts.32.370 PMC7276782

[35] Hwang HS , Park IY , Hong JI , et al. Comparison of joint degeneration and pain in male and female mice in DMM model of osteoarthritis. Osteoarthritis Cartilage 2021;29:728–38. 33609695 10.1016/j.joca.2021.02.007

[36] Parmelee PA , Tighe CA , Dautovich ND . Sleep disturbance in osteoarthritis: linkages with pain, disability, and depressive symptoms. Arthritis Care Res (Hoboken) 2015;67:358–65. 25283955 10.1002/acr.22459 PMC4342277

[37] Gomis A , Meini S , Miralles A , et al. Blockade of nociceptive sensory afferent activity of the rat knee joint by the bradykinin B2 receptor antagonist fasitibant. Osteoarthritis Cartilage 2013;21:1346–54. 23973149 10.1016/j.joca.2013.03.013

[38] Chakrabarti S , Ai M , Wong K , et al. Functional characterization of ovine dorsal root ganglion neurons reveal peripheral sensitization after osteochondral defect. eNeuro 2021;8:ENEURO.0237‐21.2021. 10.1523/ENEURO.0237-21.2021 PMC8577045 34544757

[39] Kelly S , Dobson KL , Harris J . Spinal nociceptive reflexes are sensitized in the monosodium iodoacetate model of osteoarthritis pain in the rat. Osteoarthritis Cartilage 2013;21:1327–35. 23973147 10.1016/j.joca.2013.07.002

[40] Chakrabarti S , Hore Z , Pattison LA , et al. Sensitization of knee‐innervating sensory neurons by tumor necrosis factor‐α‐activated fibroblast‐like synoviocytes: an in vitro, coculture model of inflammatory pain. Pain 2020;161:2129–41. 32332252 10.1097/j.pain.0000000000001890 PMC7431145

[41] Zhang YH , Vasko MR , Nicol GD . Ceramide, a putative second messenger for nerve growth factor, modulates the TTX‐resistant Na+ current and delayed rectifier K+ current in rat sensory neurons. J Physiol 2002;544:385–402. 12381813 10.1113/jphysiol.2002.024265 PMC2290585

[42] Miller RE , Tran PB , Das R , et al. CCR2 chemokine receptor signaling mediates pain in experimental osteoarthritis. Proc Natl Acad Sci 2012;109:20602–7. 23185004 10.1073/pnas.1209294110 PMC3528555

[43] Ishihara S , Obeidat AM , Wokosin DL , et al. The role of intra‐articular neuronal CCR2 receptors in knee joint pain associated with experimental osteoarthritis in mice. Arthritis Res Ther 2021;23:103. 33827672 10.1186/s13075-021-02486-y PMC8025346

