# Supplementary material for: Role of Human Mesenchymal Stem Cells and Derived Extracellular Vesicles in Reducing Sensory Neuron Hyperexcitability and Pain Behaviors in Murine Osteoarthritis
Source: Arthritis Rheumatol. 2022 Dec 28;75(3):352–63. doi: 10.1002/art.42353 (PMC10952633; doi:10.1002/art.42353)
Supplement: Supplementary file 1 — Disclosure Form [file ART-75-352-s001.pdf]

ICMJE DISCLOSURE FORM

Date: 22.07.22
Your Name: Minji Ai
Manuscript Title: *Human mesenchymal stem cells and derived extracellular vesicles reduce sensory neuron hyperexcitability and pain behaviors in murine osteoarthritis*
Manuscript number (if known): ar-22-0584

In the interest of transparency, we ask you to disclose all relationships/activities/interests listed below that are related to the content of your manuscript. "Related" means any relation with for-profit or not-for-profit third parties whose interests may be affected by the content of the manuscript. Disclosure represents a commitment to transparency and does not necessarily indicate a bias. If you are in doubt about whether to list a relationship/activity/interest, it is preferable that you do so.

The following questions apply to the author's relationships/activities/interests as they relate to the current manuscript only.

The author's relationships/activities/interests should be defined broadly. For example, if your manuscript pertains to the epidemiology of hypertension, you should declare all relationships with manufacturers of antihypertensive medication, even if that medication is not mentioned in the manuscript.

In item #1 below, report all support for the work reported in this manuscript without time limit. For all other items, the time frame for disclosure is the past 36 months.

		**Name all entities with whom you have this relationship or indicate none (add rows as needed)**	**Specifications/Comments (e.g., if payments were made to you or to your institution)**
		**Time frame: Since the initial planning of the work**	
1	All support for the present manuscript (e.g., funding, provision of study materials, medical writing, article processing charges, etc.) **No time limit for this item.**	None	
		**Time frame: past 36 months**	
2	Grants or contracts from any entity (if not indicated in item #1 above).	None	
3	Royalties or licenses	None	
4	Consulting fees	None	

5	Payment or honoraria for lectures, presentations, speakers bureaus, manuscript writing or educational events	None	
6	Payment for expert testimony	None	
7	Support for attending meetings and/or travel	None	
8	Patents planned, issued or pending	None	
9	Participation on a Data Safety Monitoring Board or Advisory Board	None	
10	Leadership or fiduciary role in other board, society, committee or advocacy group, paid or unpaid	None	
11	Stock or stock options	None	
12	Receipt of equipment, materials, drugs, medical writing, gifts or other services	None	
13	Other financial or non-financial interests	None	

Please place an "X" next to the following statement to indicate your agreement:

X I certify that I have answered every question and have not altered the wording of any of the questions on this form.

ICMJE DISCLOSURE FORM

Date: 22.07.22
Your Name: William E. Hotham
Manuscript Title: *Human mesenchymal stem cells and derived extracellular vesicles reduce sensory neuron hyperexcitability and pain behaviors in murine osteoarthritis*
Manuscript number (if known): ar-22-0584

In the interest of transparency, we ask you to disclose all relationships/activities/interests listed below that are related to the content of your manuscript. "Related" means any relation with for-profit or not-for-profit third parties whose interests may be affected by the content of the manuscript. Disclosure represents a commitment to transparency and does not necessarily indicate a bias. If you are in doubt about whether to list a relationship/activity/interest, it is preferable that you do so.

The following questions apply to the author's relationships/activities/interests as they relate to the current manuscript only.

The author's relationships/activities/interests should be defined broadly. For example, if your manuscript pertains to the epidemiology of hypertension, you should declare all relationships with manufacturers of antihypertensive medication, even if that medication is not mentioned in the manuscript.

In item #1 below, report all support for the work reported in this manuscript without time limit. For all other items, the time frame for disclosure is the past 36 months.

		**Name all entities with whom you have this relationship or indicate none (add rows as needed)**	**Specifications/Comments (e.g., if payments were made to you or to your institution)**
**Time frame: Since the initial planning of the work**			
1	All support for the present manuscript (e.g., funding, provision of study materials, medical writing, article processing charges, etc.) **No time limit for this item.**	None	
**Time frame: past 36 months**			
2	Grants or contracts from any entity (if not indicated in item #1 above).	None	
3	Royalties or licenses	None	
4	Consulting fees	None	

5	Payment or honoraria for lectures, presentations, speakers bureaus, manuscript writing or educational events	None	
6	Payment for expert testimony	None	
7	Support for attending meetings and/or travel	None	
8	Patents planned, issued or pending	None	
9	Participation on a Data Safety Monitoring Board or Advisory Board	None	
10	Leadership or fiduciary role in other board, society, committee or advocacy group, paid or unpaid	None	
11	Stock or stock options	None	
12	Receipt of equipment, materials, drugs, medical writing, gifts or other services	None	
13	Other financial or non-financial interests	None	

Please place an "X" next to the following statement to indicate your agreement:

X I certify that I have answered every question and have not altered the wording of any of the questions on this form.

ICMJE DISCLOSURE FORM

Date: 22.07.22
Your Name: Luke Pattison
Manuscript Title: *Human mesenchymal stem cells and derived extracellular vesicles reduce sensory neuron hyperexcitability and pain behaviors in murine osteoarthritis*
Manuscript number (if known): ar-22-0584

In the interest of transparency, we ask you to disclose all relationships/activities/interests listed below that are related to the content of your manuscript. "Related" means any relation with for-profit or not-for-profit third parties whose interests may be affected by the content of the manuscript. Disclosure represents a commitment to transparency and does not necessarily indicate a bias. If you are in doubt about whether to list a relationship/activity/interest, it is preferable that you do so.

The following questions apply to the author's relationships/activities/interests as they relate to the current manuscript only.

The author's relationships/activities/interests should be defined broadly. For example, if your manuscript pertains to the epidemiology of hypertension, you should declare all relationships with manufacturers of antihypertensive medication, even if that medication is not mentioned in the manuscript.

In item #1 below, report all support for the work reported in this manuscript without time limit. For all other items, the time frame for disclosure is the past 36 months.

		**Name all entities with whom you have this relationship or indicate none (add rows as needed)**	**Specifications/Comments (e.g., if payments were made to you or to your institution)**
		**Time frame: Since the initial planning of the work**	
1	All support for the present manuscript (e.g., funding, provision of study materials, medical writing, article processing charges, etc.) **No time limit for this item.**	None	
		**Time frame: past 36 months**	
2	Grants or contracts from any entity (if not indicated in item #1 above).	None	
3	Royalties or licenses	None	
4	Consulting fees	None	

5	Payment or honoraria for lectures, presentations, speakers bureaus, manuscript writing or educational events	None	
6	Payment for expert testimony	None	
7	Support for attending meetings and/or travel	None	
8	Patents planned, issued or pending	None	
9	Participation on a Data Safety Monitoring Board or Advisory Board	None	
10	Leadership or fiduciary role in other board, society, committee or advocacy group, paid or unpaid	None	
11	Stock or stock options	None	
12	Receipt of equipment, materials, drugs, medical writing, gifts or other services	None	
13	Other financial or non-financial interests	None	

Please place an "X" next to the following statement to indicate your agreement:

X I certify that I have answered every question and have not altered the wording of any of the questions on this form.

ICMJE DISCLOSURE FORM

Date: 22.07.22
Your Name: Qingxi Ma
Manuscript Title: *Human mesenchymal stem cells and derived extracellular vesicles reduce sensory neuron hyperexcitability and pain behaviors in murine osteoarthritis*
Manuscript number (if known): ar-22-0584

In the interest of transparency, we ask you to disclose all relationships/activities/interests listed below that are related to the content of your manuscript. "Related" means any relation with for-profit or not-for-profit third parties whose interests may be affected by the content of the manuscript. Disclosure represents a commitment to transparency and does not necessarily indicate a bias. If you are in doubt about whether to list a relationship/activity/interest, it is preferable that you do so.

The following questions apply to the author's relationships/activities/interests as they relate to the current manuscript only.

The author's relationships/activities/interests should be defined broadly. For example, if your manuscript pertains to the epidemiology of hypertension, you should declare all relationships with manufacturers of antihypertensive medication, even if that medication is not mentioned in the manuscript.

In item #1 below, report all support for the work reported in this manuscript without time limit. For all other items, the time frame for disclosure is the past 36 months.

		**Name all entities with whom you have this relationship or indicate none (add rows as needed)**	**Specifications/Comments (e.g., if payments were made to you or to your institution)**
		**Time frame: Since the initial planning of the work**	
1	All support for the present manuscript (e.g., funding, provision of study materials, medical writing, article processing charges, etc.) **No time limit for this item.**	None	
		**Time frame: past 36 months**	
2	Grants or contracts from any entity (if not indicated in item #1 above).	None	
3	Royalties or licenses	None	
4	Consulting fees	None	

5	Payment or honoraria for lectures, presentations, speakers bureaus, manuscript writing or educational events	None	
6	Payment for expert testimony	None	
7	Support for attending meetings and/or travel	None	
8	Patents planned, issued or pending	None	
9	Participation on a Data Safety Monitoring Board or Advisory Board	None	
10	Leadership or fiduciary role in other board, society, committee or advocacy group, paid or unpaid	None	
11	Stock or stock options	None	
12	Receipt of equipment, materials, drugs, medical writing, gifts or other services	None	
13	Other financial or non-financial interests	None	

Please place an "X" next to the following statement to indicate your agreement:

X I certify that I have answered every question and have not altered the wording of any of the questions on this form.

ICMJE DISCLOSURE FORM

Date: 22.07.22
Your Name: Frances M. D. Henson
Manuscript Title: *Human mesenchymal stem cells and derived extracellular vesicles reduce sensory neuron hyperexcitability and pain behaviors in murine osteoarthritis*
Manuscript number (if known): ar-22-0584

In the interest of transparency, we ask you to disclose all relationships/activities/interests listed below that are related to the content of your manuscript. "Related" means any relation with for-profit or not-for-profit third parties whose interests may be affected by the content of the manuscript. Disclosure represents a commitment to transparency and does not necessarily indicate a bias. If you are in doubt about whether to list a relationship/activity/interest, it is preferable that you do so.

The following questions apply to the author's relationships/activities/interests as they relate to the current manuscript only.

The author's relationships/activities/interests should be defined broadly. For example, if your manuscript pertains to the epidemiology of hypertension, you should declare all relationships with manufacturers of antihypertensive medication, even if that medication is not mentioned in the manuscript.

In item #1 below, report all support for the work reported in this manuscript without time limit. For all other items, the time frame for disclosure is the past 36 months.

		**Name all entities with whom you have this relationship or indicate none (add rows as needed)**	**Specifications/Comments (e.g., if payments were made to you or to your institution)**
		**Time frame: Since the initial planning of the work**	
1	All support for the present manuscript (e.g., funding, provision of study materials, medical writing, article processing charges, etc.) **No time limit for this item.**	Horizon 2020	Funding of materials for the project, grant number RG90905
		Innovate UK	Funding of materials for the project, grant number RG87266
		**Time frame: past 36 months**	
2	Grants or contracts from any entity (if not indicated in item #1 above).	None	
3	Royalties or licenses	None	
4	Consulting fees	None	

5	Payment or honoraria for lectures, presentations, speakers bureaus, manuscript writing or educational events	None	
6	Payment for expert testimony	None	
7	Support for attending meetings and/or travel	None	
8	Patents planned, issued or pending	None	
9	Participation on a Data Safety Monitoring Board or Advisory Board	None	
10	Leadership or fiduciary role in other board, society, committee or advocacy group, paid or unpaid	None	
11	Stock or stock options	None	
12	Receipt of equipment, materials, drugs, medical writing, gifts or other services	None	
13	Other financial or non-financial interests	None	

Please place an "X" next to the following statement to indicate your agreement:

X I certify that I have answered every question and have not altered the wording of any of the questions on this form.

ICMJE DISCLOSURE FORM

Date: 22.07.22
Your Name: Ewan St. John Smith
Manuscript Title: *Human mesenchymal stem cells and derived extracellular vesicles reduce sensory neuron hyperexcitability and pain behaviors in murine osteoarthritis*
Manuscript number (if known): ar-22-0584

In the interest of transparency, we ask you to disclose all relationships/activities/interests listed below that are related to the content of your manuscript. "Related" means any relation with for-profit or not-for-profit third parties whose interests may be affected by the content of the manuscript. Disclosure represents a commitment to transparency and does not necessarily indicate a bias. If you are in doubt about whether to list a relationship/activity/interest, it is preferable that you do so.

The following questions apply to the author's relationships/activities/interests as they relate to the current manuscript only.

The author's relationships/activities/interests should be defined broadly. For example, if your manuscript pertains to the epidemiology of hypertension, you should declare all relationships with manufacturers of antihypertensive medication, even if that medication is not mentioned in the manuscript.

In item #1 below, report all support for the work reported in this manuscript without time limit. For all other items, the time frame for disclosure is the past 36 months.

		**Name all entities with whom you have this relationship or indicate none (add rows as needed)**	**Specifications/Comments (e.g., if payments were made to you or to your institution)**
		**Time frame: Since the initial planning of the work**	
1	All support for the present manuscript (e.g., funding, provision of study materials, medical writing, article processing charges, etc.) **No time limit for this item.**	Versus Arthritis	Funding of materials for the project, grant number RG21973
		**Time frame: past 36 months**	
2	Grants or contracts from any entity (if not indicated in item #1 above).	None	
3	Royalties or licenses	None	
4	Consulting fees	None	

5	Payment or honoraria for lectures, presentations, speakers bureaus, manuscript writing or educational events	None	
6	Payment for expert testimony	None	
7	Support for attending meetings and/or travel	None	
8	Patents planned, issued or pending	None	
9	Participation on a Data Safety Monitoring Board or Advisory Board	None	
10	Leadership or fiduciary role in other board, society, committee or advocacy group, paid or unpaid	None	
11	Stock or stock options	None	
12	Receipt of equipment, materials, drugs, medical writing, gifts or other services	None	
13	Other financial or non-financial interests	None	

Please place an "X" next to the following statement to indicate your agreement:

X I certify that I have answered every question and have not altered the wording of any of the questions on this form.
