# Supplementary material for: Role of Human Mesenchymal Stem Cells and Derived Extracellular Vesicles in Reducing Sensory Neuron Hyperexcitability and Pain Behaviors in Murine Osteoarthritis
Source: Arthritis Rheumatol. 2022 Dec 28;75(3):352–63. doi: 10.1002/art.42353 (PMC10952633; doi:10.1002/art.42353)
Supplement: Supplementary file 2 — Appendix S1: Supplementary Information [file ART-75-352-s002.zip › [Ai] ART_42353_suppl_figures.docx]

**Supplementary Figures**


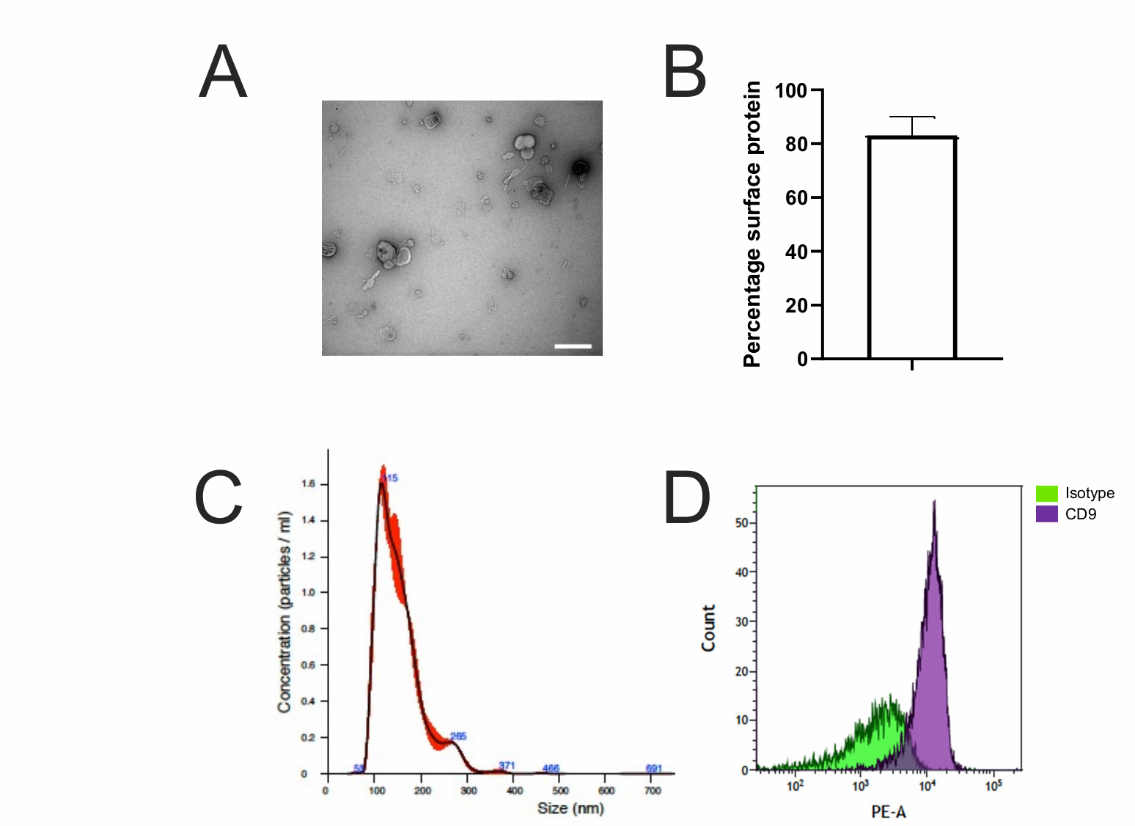


**Figure S1 Characterization of MSC-EVs. (**A) Representative image of MSC-EVs viewed with a transmission electron microscope, scale bar: 500 nm. **(**B) Percentage of MSC-EV surface protein. (C) Size distribution of MSC-EVs. Blue numbers indicate the mean particle size at the peak. Red band represent SEM range. (D) Positive signal of surface marker CD9 on MSC-EVs.


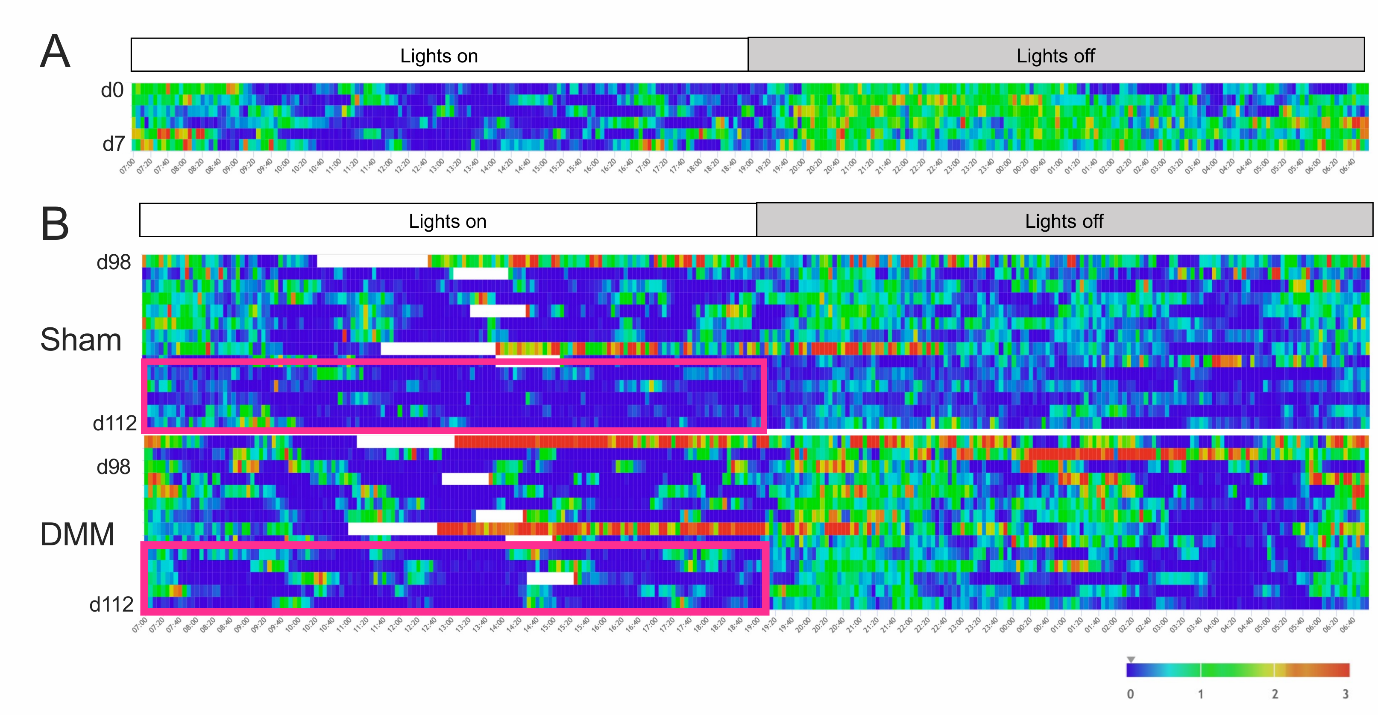


**Figure S2 Mouse activity monitored by DVC.** (A) Heatmap activity recorded from 3 experimental mice during a week prior than DMM or Sham surgery. Each colored block represents average activities recorded in 5 minutes. The 0-3 scale indicates activity values computed by extruding capacitance change. (B) Heatmap activity of sham and DMM mice from week 14 to week 16 after surgery. d98 and d112 refer to day 98 and day 112 post-surgery. White bars indicate when mice were removed from the cages for experimental procedures or behavioral tests and thus no data were recorded. The purple box shows irregular activity sprouts in DMM mice but not sham mice at week 16. Lights on period: 7:00 – 19:00; Lights off period: 19:00 – 7:00.


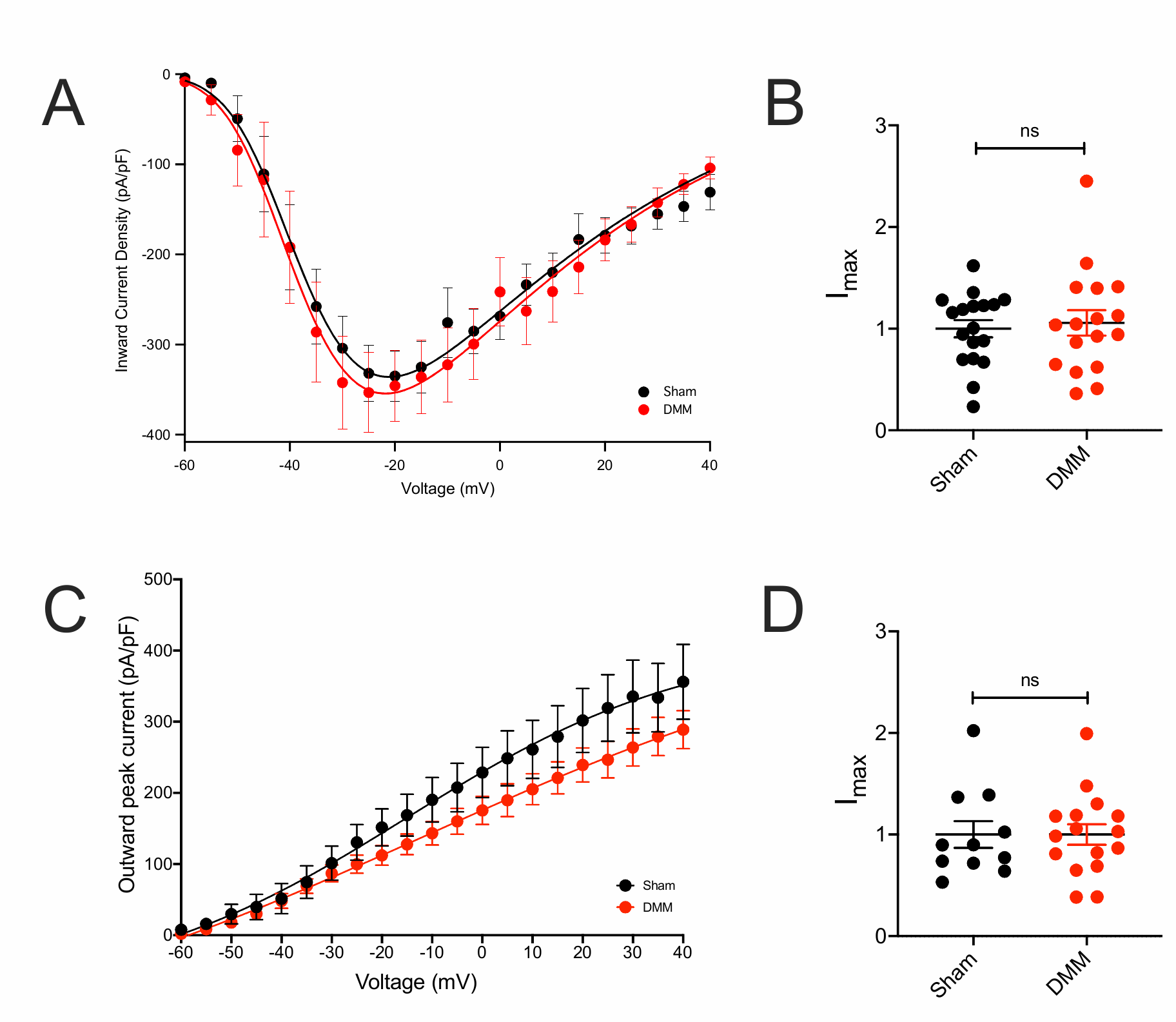


**Figure S3 Voltage-gated macroscopic currents of FB neurons.** Plots of inward (A) and outward (B) current of FB labelled DRG neurons at different voltage steps normalized by cell capacitance. Peak inward (B) and outward current (D) normalized by maximum current density in sham FB neurons. ns, no significant difference. Unpaired t test.


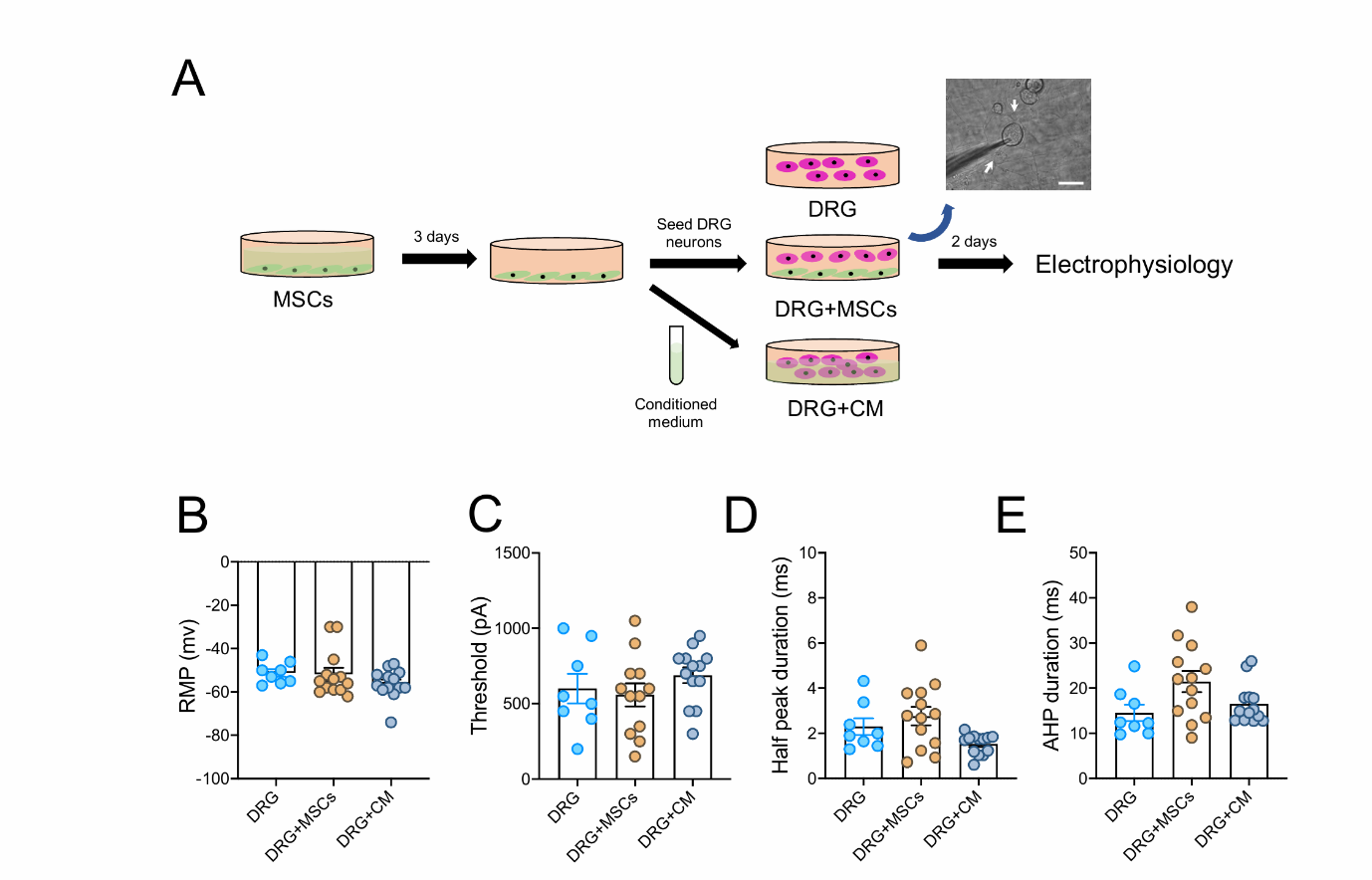


**Figure S4 MSCs and MSC conditioned medium does not affect untreated mouse DRG neuron excitability *in vitro*.** (A) Experimental design of study to investigate the impact of MSCs and MSC conditioned medium on DRG neuron excitability. Top right image, representative image of DRG neurons cocultured with MSCs. Scale bar: 50μm. (B) Resting membrane potential (RMP) mouse DRG neurons under different culture conditions, (C) action potential threshold (D), half peak duration (HPD) (E), and afterhyperpolarization (AHP) duration (F) of DRG neurons in different culture conditions. Neuron numbers in each group: DRG (n=8), DRG+MSCs (n = 13), DRG+CM (n = 13). Data are presented as Mean ± SEM in graph. One-way ANOVA with post-hoc Tukey’s test.
