# Supplementary material for: Role of Human Mesenchymal Stem Cells and Derived Extracellular Vesicles in Reducing Sensory Neuron Hyperexcitability and Pain Behaviors in Murine Osteoarthritis
Source: Arthritis Rheumatol. 2022 Dec 28;75(3):352–63. doi: 10.1002/art.42353 (PMC10952633; doi:10.1002/art.42353)
Supplement: Supplementary file 2 — Appendix S1: Supplementary Information [file ART-75-352-s002.zip › [Ai] ART_42353_suppl_methods.docx]

**Supplementary Methods**

***Digging assay***

Each mouse was placed in a standard individually ventilated cage (391×199×160 mm) filled with Aspen midi 8/20 wood chip bedding (LBS Biotechnology) tamped down to a depth of ∼4 cm. Each mouse was tested individually in a testing cage for 3 mins without food or water supply after 30 mins habituation in the testing room. Digging training was conducted one day before test. During training, the same digging procedure was carried twice with a 30-mins intermission in-between. All experiments were conducted between 12:30–14:30 in the same procedure room and videotaped by a camera (Sony FDR-AX53, UK). Analysis was conducted offline after the conclusion of all studies and following blinding of recordings.

***Mouse experimental procedures***

Mice were on a normal 12h light/dark cycle at set temperature (21℃) and were regularly monitored by animal technicians, as well as experimenters when undergoing procedures. All the surgical procedures and knee injections performed on mice were carried out under general anesthesia (GA) unless stated otherwise. GA was induced by 4% inhalable isoflurane (Zoties, USA) and maintained by 2.5% (v/v) isoflurane during procedures. Mice were sacrificed after 16-weeks post-surgery by CO_2_ exposure followed by cervical dislocation.

***Knee injections***

Stifle injections were performed under GA using a 10 μl syringe (Hamilton, USA) and a 30-gauge needle (Terumo AGANI, UK) through the patellar tendon.

***Destabilization of the medial meniscus (DMM) surgery***

A 3 mm incision was made parallel to the patella on the left leg to expose the stifle joint and the joint capsule was immediately opened using a 15 micro-surgical blade (Swann-Moston, UK). A 30-gauge needle (Terumo AGANI, UK) was used to bluntly dissect the fat pad and expose the medial meniscus (MM). The medial meniscotibial ligament (MMLT) anchoring the medial meniscus to the tibial plateau was carefully cut using a SM65A blade (Swann-Moston, UK). Skin incision was sutured using 6-0 Vicryl® (Ethicon, Belgium). Sham surgery was performed under the same procedure, but without damaging the MMLT.

***DRG neuron isolation and culture***

Collected DRG were placed into cold dissociation media (L-15 Medium (1×) + GlutaMAX-l (Life Technologies, UK) supplemented with 24 mM NaHCO3). Dissected DRG were enzymatically digested in prewarmed collagenase solution (1 mg/ml, 6 mg/ml Bovine serum albumin (BSA) in dissociation media, Sigma, UK) for 15 mins followed trypsin solution (1 mg/ml trypsin, 6 mg/ml Bovine serum albumin (BSA) in dissociation media, Sigma, UK) for 30 mins at 37℃ before mechanical trituration (i.e. pipetting up and down for 8 times). Following trituration, brief centrifugation (1000 rmp, 30s) was used to separate remaining DRG tissue from dissociated neurons. The resulting supernatant, containing the dissociated neurons, was collected in a separate tube, while 2 ml of culture medium was added into pelleted DRG tissues for further trituration. Trituration and brief centrifugation were repeated for 5 times until 10 ml of supernatant was collected. Collected supernatant was centrifuged at 1000 rmp for 5 mins to obtain cell pellets, which were resuspended in culture media and plated on poly-D-lysine and laminin coated glass bottomed dishes (MatTek, USA). Neurons were incubated at 37℃, 5% CO_2_ for overnight or 48-hours before electrophysiology depending on the experiments.

***Histology***

Operated knee joints were collected post-mortem and fixed in 4% (v/v) paraformaldehyde (PFA, Sigma, UK) for 24-hours prior than decalcification. Fixed samples were washed in distilled water for 30 minutes before 21 days of decalcification in 14% (v/v) ethylenediaminetetraacetic acid (EDTA, Sigma, UK) solution (pH 8, adjusted by NaOH pellets) at room temperature (21℃). The completion of decalcification was confirmed through the easy penetration of the tibia bone with a 27G needle. Decalcified joints were processed in graded ethanol series (30, 50, 75, 90, 95, 100 and 100%, 1-hour each), xylene (3×, 1.5-hour each), paraffin (3×, 2-hours each) (Fisher, UK) in tissue processor (Leica TP1020 tissue processor, UK) and embedded in paraffin using embedding station (Leica HistoCore Arcadia H embedding station, UK) following routine histological procedures. Embedded samples were sectioned to 7 μm sections using a microtome (Leica RM2235, UK), and mounted on HistoBond slides (StatLab, UK). Slides were deparaffinized and hydrated before staining. Slides were first heated at 60℃ for 10 mins following three sequential xylene baths (5mins each), an increased series of ethanol solution (100%, 100%, 95%, 80%, 70%, 50%, 30%; 3 mins each) and distilled water (5 mins) before staining. Hydrated slides were first stained with Weight’s Iron Hematoxylin (Sigma, UK) working solution 7 mins and gently washed with running tap water for 10 mins to remove excessive stain, followed by 3 mins stain with 0.08% (w/v) fast green FCF (Sigma, UK), 10s 1% (w/v) Acetic acid, and 5 mins 0.1% (w/v) Safranin O (Sigma) before a single dip in 0.5% (w/v) Acetic acid. Slides were then briefly dehydrated with 100% ethanol (2 mins), cleared in xylene (2 mins) and mounted with ProLong® Gold Antifade Mountant (ThermoFisher, UK). Mounted slides were scanned by a PerciPoint O8 microscope and imaged by corresponding ViewPoint software (PerciPoint, Germany).

***Electrophysiology***

DRG neurons were bathed in extracellular solution (ECS) (in mM): NaCl (140), KCl (4), CaCl_2_ (2), MgCl_2_ (1), glucose (4), HEPES (10), adjusted to pH 7.4 with NaOH, and osmolarity was adjusted to 280-295 mOsm by sucrose) and recorded by an EPC-10 amplifier (HEKA, Germany) with corresponding software Patchmaster. Patch glass pipettes (4-9 MΩ, Hilgenberg) were pulled by a P-97 Flaming/Brown puller (Sutter Instruments, USA) from borosilicate glass capillaries and loaded with intracellular solution (ICS) (in mM)—KCl (110), NaCl (10), MgCl_2_ (1), EGTA (1), and HEPES (10), adjusted to pH 7.3 with KOH (300-310 mOsm). Ground electrode was placed in the bath to form a closed electric circuit. Fast blue labelled neurons were identified by LED excitation at 365 nm (Cairn Research, UK) with a 450/30× filter tube. Pipette and cell membrane capacitance were compensated by Patchmaster macros and series resistance was compensated by >70%. Step current (100 pA to 1000 pA) for 80 ms through 50 steps or no current were injected to generate action potential (AP) under current-clamp mode. AP threshold, half peak duration (HPD, ms), and afterhyperpolarization duration (AHP, ms) and amplitude (mV), were measured in FitMaster (HEKA, Germany) software as previous described (28). Voltage-sensitive ion channel activities were assessed under voltage-clamp mode with leak subtraction and series compensation. Cells were held at -120 mV for 240 ms before stepping to the test potential (-60 mV to 50 mV in 5 mV increments) for 40 ms and returned to holding potential (-60 mV) for 200 ms between sweeps. Peak inward and outward voltage-gated current density (pA/pF) were calculated by maximum current (normalized by subtracting average baseline amplitude (5s)) amplitude dividing cell capacitance. Voltage-current relationships were fitted in IgorPro software (Wavemetrics, USA) using the following Boltzmann equation to determine reversal potential (E_rev_) and the half peak activation potential (V_half_):

$$f(x)= \Gamma\times x \times\frac{1-e^{-\frac{x-E_{\mathrm{rev}}}{25mV}}}{1-e^{-\frac{x}{25mV}}} \times\frac{1}{{(1+e^{-\frac{x-V_{\mathrm{half}}}{\mathrm{slope}}})}^{3}}$$

where Γ is the constant, and x is the command potential. To compare the size of current density among neuron groups, the maximum inward or outward current density was normalized to those obtained from the sham neuron with maximum current as I_max_.

***Extracellular vesicle isolation***

Extracellular vesicles were harvested based on previous description (*36*). MSCs were cultured in standard cell culture media α-MEM (Thermo, UK) supplemented with 10% v/v fetal calf serum (thermo, UK), 1% (v/v) Glutamax (100×) (Gibco, UK), 1% (v/v) P/S (Gibco, UK), and incubated at 37 °C, 5% CO_2_. Passage three MSCs at 80% confluence were switched to serum free culture medium (α-MEM (Thermo, UK), 1% (v/v) Glutamax (100×) (Gibco, UK), 1% (v/v) P/S (Gibco, UK)) for 48-hours incubation. The conditioned medium was then collected and centrifuged at 300 g for 5 minutes, with supernatant transferred to a falcon tube for further centrifugation at 2,000 g for 20 minutes at 4℃. Cell numbers were counted by a hemocytometer. Supernatant was then transferred into polycarbonate ultracentrifuge tubes (Beckman, USA) for differential sequential ultracentrifugation at 10,000 g for 45 minutes and 100,000 g for 90 minutes. Collected pellet was resuspended in PBS for a further ultracentrifugation at 100,000 g for 90 minutes. Newly collected pellet was resuspended in 1ml PBS and stored at -70℃ for use.

***Nanoparticle Tracking Analysis***

Collected MSC-EVs sample was diluted 1:50 in PBS for Nanoparticle Tracking Analysis (NTA, Malvern, UK). Sample was further diluted from 1:100 to 1:500 with density over 50 particles/frame. Diluted sample was loaded into a NanoSight LM10 Nanoparticle Analysis system following manufacturer’s instruction with a syringe pump rate of 1,000 (Arbitrary units). The analysis was performed in NTA 1.4 analytical software.

***BCA assay***

Total surface protein content of MSC-EVs was measured by the Pierce BCA Protein Assay Kit following manufacturer's instructions (Thermo scientific, UK).

***Transmission electron microscope (TEM)***

The MSC-EV suspension was placed on ‘Glow discharge disks’ pre-prepared by the Cambridge Electron Microscopy group. The samples were negatively stained with 2% uranyl acetate in PBS (Sigma, USA) for 2 minutes followed by twice PBS wash and viewed under TEM. Images were acquired by an ORCA HR high resolution CCD camera with a Hamamatsu DCAM board running Image Capture Engine software, version 600.323 (Advanced Microscopy Technology Corp., Danvers, MA, USA).

***Flow cytometry***

MSC-EVS were conjugated to 1 μl of 4% aldehyde/sulphate latex beads (Invitrogen, UK) by overnight incubation on a rotary wheel at room temperature with 1ml PBS. 110 μl of 2 M glycine (Sigma, USA) was added following the overnight incubation step (final concentration 200 mM) for 30 minutes before centrifugation at 3,000g for 5 minutes. The sample pellet was resuspended in 1 ml of 0.5% (v/v) FCS in PBS following supernatant removal. Same centrifugation step was applied with pellet was re-suspended in 50 μl of 0.5% (v/v) FCS in PBS afterwards. Resuspended sample was then stained with 1 μl PE anti-human CD9 Antibody (Biologend, UK) at 4 ℃ for 20 minutes before being diluted in 3ml of 0.5% (v/v) FCS in PBS, centrifuged at 3,000g, and resuspended in 300 μl PBS. Fluorochrome compensation control was prepared by adding one drop of OneComp eBeads (eBioscience, UK) and 0.5 μl of tested antibodies with distinct fluorochrome into 200 μl 0.5% (v/v) FCS in PBS. Prepared samples were stored on ice and scanned by a BD FACS Canto II flow cytometry analyzer (BD Bioscience, UK) within 30 minutes after preparation. Analysis was performed in Kaluza software (Beckman coulter life science, USA) with corrected overlap emission through single stained compensation controls. Only single and live cells were gated during the analysis.

***In vitro* coculture of MSCs and naïve mouse DRG neurons**

MSCs were cultured for 3 days to obtain MSC secretome conditioned medium (CM). DRG neurons were then isolated from naïve mice and cultured alone, in the presence of MSCs, or with CM for 40-48h. Electrophysiology was then performed on DRG neurons.
